# Recent Advancement in Biofluid-Based Glucose Sensors Using Invasive, Minimally Invasive, and Non-Invasive Technologies: A Review

**DOI:** 10.3390/nano12071082

**Published:** 2022-03-25

**Authors:** Vundrala Sumedha Reddy, Bhawana Agarwal, Zhen Ye, Chuanqi Zhang, Kallol Roy, Amutha Chinnappan, Roger J. Narayan, Seeram Ramakrishna, Rituparna Ghosh

**Affiliations:** 1 Centre for Nanotechnology & Sustainability, Department of Mechanical Engineering, National University of Singapore, Singapore 119260, Singapore; sumedha@u.nus.edu (V.S.R.); e0816501@u.nus.edu (Z.Y.); e0816507@u.nus.edu (C.Z.); mpecam@nus.edu.sg (A.C.); 2 Department of Chemical Engineering, BITS Pilani-Hyderabad Campus, Hyderabad 500078, India; f20171321h@alumni.bits-pilani.ac.in; 3 Centre for Advanced 2D Materials, National University of Singapore, Singapore 117546, Singapore; c2dkalr@nus.edu.sg; 4 Joint Department of Biomedical Engineering, North Carolina State University, Raleigh, NC 27695, USA; roger_narayan@unc.edu

**Keywords:** glucose detection, biosensors, blood, urine, sweat, saliva, tears, interstitial fluid, breath

## Abstract

Biosensors have potentially revolutionized the biomedical field. Their portability, cost-effectiveness, and ease of operation have made the market for these biosensors to grow rapidly. Diabetes mellitus is the condition of having high glucose content in the body, and it has become one of the very common conditions that is leading to deaths worldwide. Although it still has no cure or prevention, if monitored and treated with appropriate medication, the complications can be hindered and mitigated. Glucose content in the body can be detected using various biological fluids, namely blood, sweat, urine, interstitial fluids, tears, breath, and saliva. In the past decade, there has been an influx of potential biosensor technologies for continuous glucose level estimation. This literature review provides a comprehensive update on the recent advances in the field of biofluid-based sensors for glucose level detection in terms of methods, methodology and materials used.

## 1. Introduction

**Figure 1:**
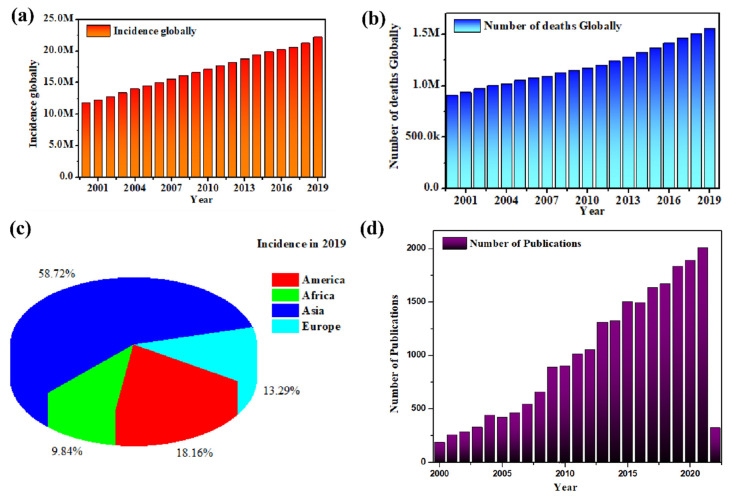
Statistical distribution of diabetic incidence around the world in recent decades. Global (**a**) incidence and (**b**) deaths since 2000. (**c**) Continent-wise distribution of diabetes incidence in 2019. Source: Global health database, February 2022. (**d**) Number of research articles published on various types of glucose sensors since 2000. Source: Scopus database, February 2022.

Diabetes is the seventh leading cause of death worldwide as estimated by the World Health Organization (WHO) in 2016. Premature mortality due to diabetes has been shooting up day by day. The total number of deaths globally reached about 1.6 million in 2019. WHO has also stated the fact that diabetes is a major cause of many health complications such as blindness, kidney failure, and heart attacks, strokes and numbness [1]. The incidence of diabetes globally has steadily increased, from 12 million to 20 million over a span of 20 years (Figure 1a). With this increasing incidence, the number of global deaths has doubled, from 0.8 million (in 2000) to 1.6 million (in 2019) in the past two decades (Figure 1b). It has also been reported that poor lifestyle choices as well as hereditary issues have a direct impact on diabetes occurrence [1]. In a recent scientific report, Lin et al. provided an analysis and forecast of the global, regional, and national burden and trends of diabetes in 195 countries and territories from 1990 to 2025. This report stated that the global incidence, prevalence, deaths, and disability-adjusted life-years associated with diabetes were at 22.9 million, 476.0 million, 1.37 million, and 67.9 million in 2017, and are projected to reach 26.6 million, 570.9 million, 1.59 million, and 79.3 million by 2025, respectively [2]. By continent, Asia is the region most seriously affected by diabetes, accounting for 58.72% of total global incidence, followed by America with 18.16%, then Europe and Africa with 13.29% and 9.84%, respectively (Figure 1c) [3]. In the past few decades, continuous research has been carried out to develop advanced diabetes detection, monitoring and prevention techniques using biomarkers to reduce the severity of the disease and improve patient quality of life. It was observed that the number of diabetes-related research articles has increased by three-fold in just the last decade (Figure 1d). The increasing global incidence of diabetes and levels of corresponding research on the development of new device solutions are directly impacting the global market for glucose monitoring devices, valued at USD 1.94 billion in 2020. This market is expected to reach USD 2.63 billion in value with a compound annual growth rate (CAGR) (of 6.24% between 2020 and 2025 [4]).

Although diabetes has no permanent cure, timely monitoring of body glucose levels and appropriate medication can hinder the complications and reduce the severity of this disease. Some of the complications in hyperglycemia (a condition where excessive glucose is circulating in the blood) involve vomiting, excessive hunger and thirst, rapid heartbeat, vision problems and others [5], whereas hypoglycemia (low blood glucose content) is accompanied by a lack of coordination, sweating, blurred vision, headache, and confusion [6]. Diabetes treatment usually includes an insulin injection or oral intake of glucose-maintaining drugs. These treatments can be made more effective by continuous monitoring of body glucose levels and appropriate medication dosages. Even though medication can regulate diabetes, overdoses can result in falling glucose levels, leading to loss of consciousness or death in severe cases. These issues have necessitated the development of advanced and reliable detection and monitoring systems that have the ability to make the diabetic patient’s life easier [7].

To make continuous glucose monitoring easier, safer, more accurate and less painful, glucose content in different biological markers (such as blood, sweat, urine, interstitial fluids, tears, breath, and saliva) has been used to corelate with blood glucose levels for years. Some of these techniques have shown promising linear relationships [8]. The generated response in turn has been used for developing calibration curves for analyte estimation [9]. The most important factors that need to be optimized for real-life applications of these sensors are linearity (the range in which the calibration curve is linear), specificity or selectivity (to avoid interference from other substrates in the sample with the detected concentration of analyte), sensitivity (electrode response to the analyte concentration) and response time (time to obtain 95% response).

**Figure 2:**
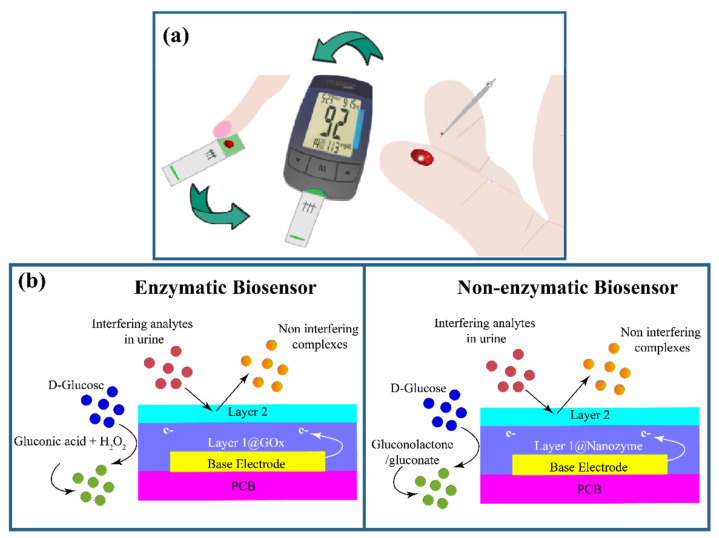
(**a**) Illustration of finger pick test. (**b**) Schematic representation of working mechanisms in enzymatic and non-enzymatic biosensors.

Typically, if the plasma glucose level is more than 200 mg/dL or fasting plasma glucose level is higher than 126 mg/dL, it is considered as a definite indication of hyperglycemia. Although a fasting blood glucose level of more than 100 mg/dL and less than 126 mg/dL is not diabetic, it is considered to be in the prediabetic stage [10]. In the majority of home devices for blood glucose measurement, testing is performed by using finger prick sticks (illustrated in Figure 2a), which is highly inconvenient for regular monitoring and also requires that relatively large amounts of blood be extracted. Even though the finger prick test requires only a few drops of blood each time, it still entails pricking of the fingers, which is painful, and the repetitive process can easily cause infections, especially in elderly and diabetic patients whose wound healing rates are slow [11]. However, traditional or standard methods of glucose level testing involve drawing at least 1 ml of blood, which is then tested using a Yellow Springs Instrument (YSI) analytical instrument to quantify the actual blood glucose concentration [12,13]. This reflects significant technological developments in this field.

In general, based on the working principle, glucose sensors can be sub-divided into the enzymatic type (involving enzymatic reaction), where glucose is detected indirectly through hydrogen peroxide (H_2_O_2_) formation, and the non-enzymatic type (using enzyme mimicking metals, metal oxides or other materials), where the change in the analyte’s properties such as fluorescence, band gaps in nanozymes, or cyclic voltammetry are used for detection [14]. Apart from change in the reacting component (enzyme or enzyme mimicking), there is a significant difference in the mechanisms involved for enzymatic and non-enzymatic sensors [15]. Commonly, enzymatic glucose sensing involves the movement of electrons due to oxidation reactions between the enzyme glucose oxidase (GOx) and glucose. GOx is a typical flavin enzyme, containing flavin adenine dinucleotide (FAD) redox active coenzyme, which catalyzes glucose to gluconolactone [16]. During the reaction, the enzyme is converted to GOx (FADH2) from GOx (FAD), resulting in the generation of current or voltage changes, which are then measured by the electrodes. The typical reaction scheme is GOx (FAD) + glucose → gluconolactone + GOx (FADH2) (Figure 2b) [17]. 

To understand the mechanism of a typical non-enzymatic sensor (Figure 2b) and the principle behind glucose selectivity [18], an example of a metal oxide nanoparticle, which serves as an electrocatalyst to transfer electrons during glucose oxidation reaction, has been given here. Metal oxide nanoparticles such as Cu_2_O, which reacts with glucose, transfer electrons through the following process, where the initial step involves electro-oxidization of Cu(I) in Cu_2_O to Cu(II) species, which is further oxidized to Cu(III) species in the presence of an alkaline solution. Amidst these transformations, when glucose is oxidized to gluconic acid, Cu(II) and Cu(III) are reduced back to Cu(I), with Cu(III) acting as electron-transfer mediator [19]. Here, the glucose selectivity of such non-enzymatic sensors depends on the extent of the glucose oxidation reaction; this phenomenon was first discovered in an acidic solution [20]. The overall reaction involving metal oxide nanoparticles happens through the following scheme:

Cu_2_O + 2OH− + H_2_O → 2Cu(OH)_2_ + 2e^−^

Cu(OH)_2_ → CuO + H_2_O

CuO + OH^−^ → CuOOH + e^−^

CuOOH = glucose → Cu(OH)_2_ + gluconic acid

Non-enzymatic sensors have gained attention in the past decade because of their superiority over enzymatic sensors, which have low performance reproducibility due to their susceptibility to varying pH levels and atmospheric humidity. Other drawbacks include enzyme deformation, which leads to stability issues and higher production costs, whereas non-enzymatic sensors show higher stability and accuracy for longer time periods [21]. To overcome these drawbacks, various new and advanced technologies such as continual glucose monitoring (CGM), photo-induced electron transfer, fluorescence resonance energy transfer (FRET) and optical sensors with fluorescence have been developed over the past few years to create highly accurate and sensitive sensors for glucose detection in both minimally invasive and non-invasive ways [22]. 

**Figure 3:**
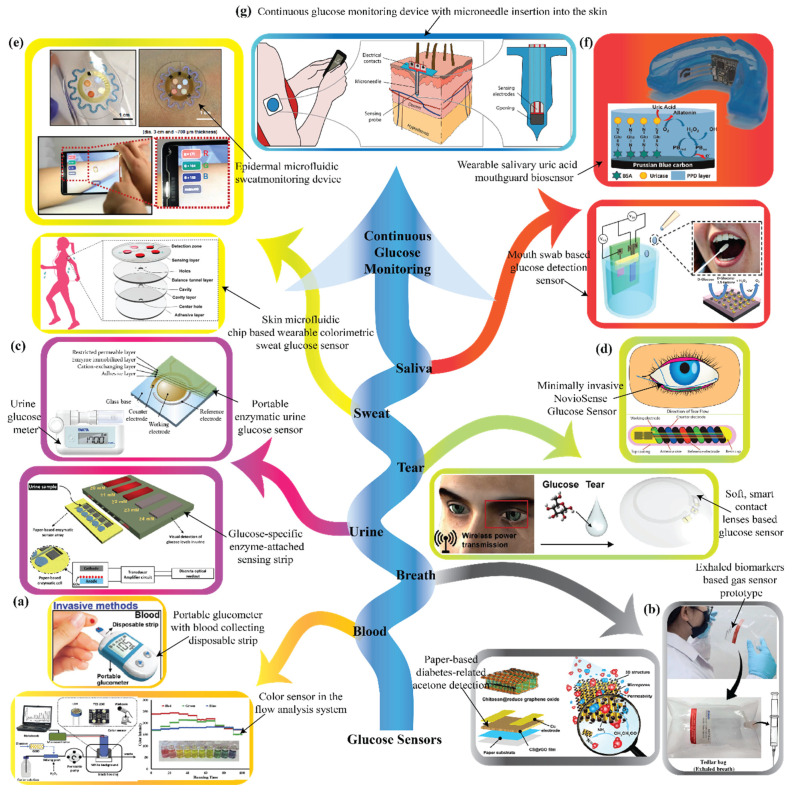
Various biomarker-based glucose sensors. (**a**) Blood glucose sensors: Portable glucometer with blood-collecting disposable strip (reproduced with permission from Lee et al., Adv. Healthc. Mater., vol. 7, no. 8, p. 1701150, 2018 [23]). The design of the color sensor in the flow analysis system. Color recognition device tested using a series solution (inset) at different pH values of 2.0 to 11.0 with the addition of universal pH solution. (Reproduced with permission from Fatoni et al., Sens. Bio-Sens. Res., pp. 30153–30159, 2020 [35]. Copyright 2020, authors under open access licenses.) (**b**) Breath glucose sensors: Paper-based diabetes-related acetone detection (Reproduced with permission from Wang et al., J. Mater. Chem. B, vol. 5, no. 22, pp. 4019–4024, 2017 [24]. Copyright 2017, Royal Society of Chemistry.) and exhaled biomarker-based gas sensor prototype. (Reproduced with permission from Xing et al., J. Mater. Chem. A, vol. 8, no. 48, pp. 26004–26012, Dec. 2020 [25]. Copyright 2020, Royal Society of Chemistry.) (**c**) Urine glucose sensors: Portable enzymatic urine glucose sensor (Reproduced with permission from N. Ito et al., Chemical, Gas, and Biosensors for Internet of Things and Related Applications, pp. 3–12, 2019 [26]. Copyright 2019, Elsevier Ltd.) and glucose-specific enzyme-attached sensing strip (Reproduced with permission from Mohammadifar et al., SLAS Technol. Transl. Life Sci. Innov., vol. 24, no. 5, pp. 499–505, Oct. 2019 [27]. Copyright 2019, authors under open access licenses.) (**d**) Tear glucose sensor: Minimally invasive NovioSense device Glucose Sensor (Reproduced with permission from Kownacka et al., Biomacromolecules 19, 11, 4504–4511, Oct. 2018 [28]. Copyright 2018, American Chemical Society.) and soft, smart contact lens-based glucose sensor (Reproduced with permission from J. Park et al., Sci. Adv., vol. 4, no. 1, p. eaap9841, Jan. 2018 [29]. Copyright 2018, authors under open access licenses.) (**e**) Sweat glucose sensors: Epidermal microfluidic sweat monitoring device (Reproduced with permission from A. Koh et al., Sci. Transl. Med., vol. 8, no. 366, pp. 366ra165, Nov. 2016 [30]. Copyright 2016, The American Association for Advancement of Science.) and layers and zones of the glucose sensor. (Reproduced with permission from Xiao et al., Anal. Chem., 2019 [36].) (**f**) Saliva glucose sensor: Wearable salivary uric acid mouthguard biosensor (Reproduced with permission from Kim et al., Biosens. Bioelectron., vol. 74, pp. 1061–1068, Dec. 2015 [32]. Copyright 2015, Elsevier Ltd.) and mouth swab-based glucose detection sensor [37]. (**g**) Intradermal continuous glucose monitoring, minimally invasive microneedle-based system. (Reproduced with permission from Ribet et al., Biomed. Microdevices, vol. 20, no. 4, p. 101, Dec. 2018 [33]. Copyright 2018, authors under open access licenses).

A large number of analyte-based biosensors have been developed and extensively commercialized for monitoring of glucose levels. A glimpse of different types of biomarker-based glucose sensors developed in past decades is depicted in Figure 3. There was a time when blood-based lab tests were being used exclusively for glucose level estimation, but the trend has shifted towards portable glucometers for direct detection using a disposable strip [23]. Various detection techniques, such as paper-based acetone sensing [24] or collection bags [25], are used for glucose detection from exhaled breath. Portable enzymatic glucometers [26] and glucose-specific enzyme-related sensing strips [27] are used for detecting glucose levels in urine. Different types of advanced optical fluid-based glucose sensors such as NovioSense from Nijmegen, Netherlands (a minimally invasive tear glucose sensor) [28] and wireless circuit-based soft lens non-invasive sensors [29] have been developed in the past 5 years. For sweat-based detection, wearable transdermal patches [30] and tattoos [31] are the most common techniques. Different types of mouth guard [32] and swab biosensors are used to detect salivary glucose levels. For severe diabetic conditions, various types of microneedle-based CGM devices have been developed which can be placed under the skin for a certain period to read and quantify the glucose level continuously for better control [33]. It was reported that in 2019, the global market for continuous glucose monitoring devices was USD 1.77 billion and is projected to reach USD 8.8 billion by 2027, which is a CAGR of 22.0% during the forecast period [34]. 

The oldest blood glucose meter was first fabricated and patented by Anton H. Clemens in the year 1971 and since then, it has come a long way, passing many hurdles, fulfilling the concept of continuous glucose monitoring [38]. This continuous glucose monitoring technique can give out readings in desired time intervals and is also facilitated by an alarm to alert patients to take appropriate action if their blood glucose levels are too high or too low [39]. The versatility of the glucose sensors has been increased by analyzing other biofluids for glucose biomarkers and correlating their levels to blood glucose levels [39]. This review covers different biofluid-based glucose sensors, while focusing on technologies ranging from the invasive and minimally invasive to non-invasive, all within the past 5 years. This progression in glucose sensors has been targeted due to the importance of continuous monitoring, point of care, ease of production and collection of biofluids, the linearity of glucose levels in the blood and other fluids, and the development and commercialization of these biofluid-based glucose sensors in the foreseeable future [40]. A scrutinized study on different working principles that exploit property-rich materials ranging from metals and metal oxides to carbon-based materials, while discussing their performance and requirements, is presented in subsequent sections. In the conclusion, we provide comprehensive information on the stand of these sensors, involving the gaps and drawbacks of each biofluid-based sensor.

## 2. Recent Developments in Various Biological Fluid-Based Glucose Sensors

Human health status and the presence of various diseases with their severity can be detected by analyzing biomarkers present in biofluids. This has made biomarkers a highly studied area in the research field, both scientifically and clinically, for health screening, diagnosis, assessments of disease recurrence and therapeutic monitoring [41]. In diabetic patients, glucose levels in other biofluids (such as urine, saliva, tears, breath sweat, and interstitial fluids) and blood are correlated to some degree. Moreover, their ease of production and collection have led to extensive research and development on biofluid-based glucose sensors, making the glucose level detection and monitoring process more patient-friendly [42].

In the determination of glucose levels in patients, blood has been used for decades. Although the use of blood is accurate and selective, with the growing number of diabetic patients and the discomfort they face with invasive methods, there has been an increased need for sophisticated, fast, and minimally invasive to non-invasive methods [43]. To address this issue, many researchers have been working on the development of sensors with capabilities to detect glucose in other biological fluids such as sweat, saliva, ocular fluid, interstitial fluid, and breath.

### 2.1. Saliva-Based Glucose Sensors

Saliva is an important biological fluid that has been employed as a tool in various toxicology and forensic medicine due to its correlation to many hormonal, immunological, metabolic and nutritional conditions of the human body. Salivary glucose is directly corelated with the blood glucose; according to the literature, a physiological glucose level within 1.0 mg/dL to 20 mg/dL is considered as hyperglycemia, whereas 0.5 mg/dL to 1.0 mg/dL range indicates a healthy individual, and levels less than 0.5 mg/dL indicate hypoglycemia [44,45]. The chemistry involved has been extensively studied for the development of different salivary biomarkers because of their ease of collection and non-invasiveness [46,47].

In the field of saliva-based glucose sensors, extensive research has been performed in both enzymatic and non-enzymatic techniques. Among various types of enzymatic sensors, organic electrochemical transistors (OECTs) have emerged as a potential glucose sensing platform. Recently, Caizhi Liao et al. developed a poly(3,4-ethylene dioxythiophene):poly(styrene sulfonate) (PEDOT:PSS) based OECT on thin polyethylene terephthalate (PET) substrates with Pt electrodes, which was used to detect and quantify H_2_O_2_ produced from oxidation of glucose by enzyme glucose oxidase (GOx). Here, selectivity was further enhanced by subsequently coating the Pt electrodes with thin graphene flakes (Nafion) followed by polyaniline (PANI) conducting polymer. This device has been proven to have a limit of detection (LOD) of 30 nM and could be used for selective detection of uric acid and cholesterol as well [48]. Following this, Elkington and co-workers further reduced the device response time to 500 s from 800 s by varying the sensor’s morphology, active layer thickness, and structure and removing the dielectric layer for rapid glucose monitoring while using a cost-effective inkjet printing technique [49]. 

**Figure 4:**
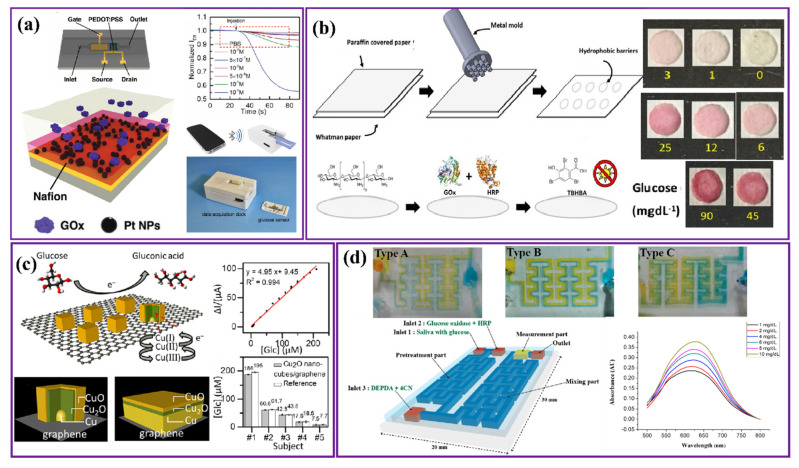
(**a**) Schematic of portable saliva glucose sensor with a thin layer of Nafion that can interact with smartphone. (Reproduced with permission from X. JI et al., Adv. Mater. Technol., vol. 1600042, pp. 1–8, 2016 [50]. Copyright 2016, John Wiley and Sons.) (**b**) Schematic representation of paper-based sensor fabrication, along with the color calibration scale for quantification of glucose concentrations in the sample. (Reproduced with permission from Dominguez et al., Sensors, vol. 18, no. 1071, pp. 1–12, 2018 [54]. Copyright 2018, authors under open access licenses.) (**c**) Mechanism involved in the sensor and schematics of Cu2O nano cube on graphene and Cu film sputter-coated on graphene. (Reproduced with permission from Gao et al., ACS Appl. Nano Mater., May 2021 [17]. Copyright 2021, authors under open access licenses.) (**d**) The proposed micro-channel with different structures in order to improve mixing efficiency. Micro-channel structures: Type A without obstacles, Type B with obstacles, Type C with obstacles and more curvature. (Reproduced with permission from Jung et al., Sensors, vol. 17, no. 2607, pp. 1–12, 2017 [55]. Copyright 2017, authors under open access licenses).

In another work, Ji et al. developed a biosensor with OECT integrated on a polydimethylsiloxane (PDMS) microfluidic channel with a conducting polymer–Nafion layer, which reduced the analyte amount and analysis time to 30 μL and 60 s, respectively. A prototype was designed for real-time sensing by linking the OECT sensor with a smartphone through Bluetooth, and exhibited 0.1 μM LOD (Figure 4a) [50]. Following this work, a layered OECT was developed with a similar setup with electrodeposited Ni/Al layered double hydroxide (LDH) for immobilizing GOx enzyme at the gate electrode. The sensor responded to glucose with high sensitivity (0.12 mA M^−1^cm^−2^) recorded in the linear range of 0.1–1.0 mM and a logarithmic range of 1–8 mM with an LOD of 0.02 mM [51].

An enzyme dendrimer-based photofluorometer with a magnetic core of iron oxide (Fe_3_O_4_) encapsulated within polyamidoamine (PAMAM) dendrimers was developed by Shende et al. Here, Fe_3_O_4_ nanoparticles were synthesized by oxidation of ferrous chloride and potassium hydroxide, whereas PAMAM dendrimers were prepared through nucleophilic addition and amidation reactions of ethylene diamine and methyl methacrylate reactants. In this study, glutaraldehyde and Tris(1, 10-phenanthroline) ruthenium (II) chloride hydrate [TPR(II)CH] were used as cross-linking agents for GOx immobilization and fluorescent indicator, respectively. Due to the magnetic nature of the sensor, the enzyme–analyte reaction can be easily washed off from the sensor surface after each use, making it reusable. However, on further investigation, it was reported that the enzymatic activity of these sensors decreased tremendously after multiple washes, which made them a less likely candidate for reusable biosensors [52]. 

From an economic point of view, a disposable, low-cost, smartphone-attachable test strip was developed from filter paper using pH-indicating dye and glucose oxidase for glucose level detection. The change in color was studied through the RGB (red green blue) profile, and an exponential relation was observed with a linear range of 50–540 mg/dL and LOD of 24.6 mg/dL. The correlation factor between blood glucose levels and salivary glucose was also determined and found to be 0.44 for non-diabetics, 0.64 for pre-diabetics and 0.94 for diabetics. An Android application was designed to study the detection zone through calculating the change in slope for red, green, and blue pixels. Additionally, the effects of various factors such as camera type, the presence of interference, etc., were studied, and ambient light was found to be a major source of error. However, to overcome this issue, a box with a hole was engineered for capturing the picture of the strip [53].

A different study involved a colorimetric bienzymatic paper-based sensor that exhibits color change in the presence of salivary glucose. The device is suitable for detection with the naked eye, while a scanner was used to obtain the quantitative value. The sensor was fabricated using paraffin-coated Whatman paper and pressed using a hot metal mold for creating the hydrophobic barriers that form the detection zone within the device. The schematic represented in Figure 4b is a simple visual of the developed methodology for the detection zone along with a color calibration for quantification of glucose concentrations in the sample. The device showed an LOD of 0.37 mg/dL within a linear glucose concentration range of 1 mg/dL to 22.5 mg/dL. Considering the simplicity of usage, absence of any sophisticated equipment, low cost, selectivity and good sensitivity, the device is suitable for the monitoring of glucose levels in low-income areas and in regions with a lack of medical facilities or professional help [54].

There has been an accelerated effort to develop non-enzymatic electrochemical salivary sensors for glucose detection due to the disadvantages associated with the stability of enzymatic sensors over long-term storage, as discussed earlier. Recently, Gao et al. reported on a nonenzymatic saliva sensor using electrodeposited Cu_2_O (cuprous oxide) nano cubes on a graphene strip that showed better performance in terms of the detection range and sensitivity compared to other Cu_2_O nanomaterial-based enzymatic and non-enzymatic glucose sensors (Figure 4c). Here, the large specific surface area of Cu_2_O nano cubes facilitates a higher number of active sites, and the graphene strip provides excellent conductivity as the substrate material. The device is capable of detecting glucose within a 0.002–17.1 mM range with 95% accuracy and the highest sensitivity of 36.40 μA mM^−1^ cm^−2^ when tested with real saliva samples. The reported device is also air-stable and can remain functional even after 6 months [17].

Keeping portability, reliability and user convenience in mind, recently a non-enzymatic lab on a chip (LOC) device was developed using microelectromechanical systems (MEMS) technology. The device was subdivided into three different zones: the pre-treatment zone, used for glucose and glucose oxidase reactions; the mixing zone, utilized for combining H_2_O_2_ and saliva with coloring agent; and the measurement zone, containing a light-emitting diode (LED) and a photodiode and used for glucose detection (Figure 4d). Here, glucose is detected through the decomposition of H_2_O_2_ into water by the horseradish peroxidase (HRP) enzyme in the presence of N,N’-diethyl-p-phenylenediamine (DEPDA) and 4-chloro-1-naphthol (4CN), imparting a blue color to the saliva, whose absorbance is measured through the photodiode. It was observed that with increasing glucose concentrations within the 0–10 mg/dL range, the absorbance of light (at a wavelength of 630 nm) in the saliva increases; hence, the output current in the photodiode decreases linearly. The device performance was further optimized by using three different types (type A, B and C) of mixing systems by varying the micro-channel structure, and it was reported that the type C mixing part, which had more curvature along with obstacles at the edges, exhibited higher sensitivity and accuracy [55]. In another study, an optical fiber-based salivary glucose detector was fabricated using an ultrasensitive long-period grating (LPG) structure where glucose transforms to glycated hemoglobin during reaction. In this process, hemoglobin is directly immobilized on the fiber structure through a matrix-assisted pulsed-laser evaporation method, resulting in spectral shifts due to the change in refractive index, which is correlated to the glucose concentration in the sample. The device can show reliable performance for glucose concentration as low as 0.1 mmol/L with an LOD of 10 μM [56].

A novel, highly sensitive, non-enzymatic salivary glucose sensor was fabricated by utilizing a large surface-to-volume ratio and high-porosity electrospun fiber by Xu et al. [44]. Here, a copper oxide nanoparticle (CuO NP) decorated polycaprolactone@polypyrrole fiber-modified indium–tin oxide (CuO/PCL@PPy/ITO) electrode was used for glucose detection. The device showed linearity within 2 µM to 6 mM glucose range with high recovery (ranging from 96.36% to 105.6%) and lowest detection limit of 0.8 µM [44]. In another study, Wang and coworkers developed a hierarchical IrO_2_@NiO (iridium oxide@ nickel oxide) structure by combining electrospinning and chemical bath deposition techniques for non-enzymatic glucose detection [57]. Here, IrO_2_ nanowires act as a conducting core, whereas NiO, which has been exploited for its sensing properties, oxidizes glucose to gluconolactone and enhances device sensitivity by providing a large surface-active area during reaction. For better entrapment in the sensing layer, the nanostructures were further covered over by glassy carbon (GC) electrodes following the Nafion polymer layer. This biosensor showed good repeatability, long-term stability, high resolution and selectivity towards glucose with high sensitivity (1439.4 μA mM^−1^ cm^−2^) in the 0.5 µM–2.5 mM linear range with an LOD of 0.31 µM [57]. 

In another study, Anderson et al. produced a non-enzymatic glucose sensor through nucleation of silver nanoparticles (AgNPs) on molybdenum disulfide (MoS_2_) [58]. Here, AgNPs were used to increase the sensitivity of the sensor, whereas MoS_2_ provided intercalation sites for the reaction. Square wave voltammetry, owing to its high sensitivity and speed, was used for glucose detection. The saliva samples were mixed with sodium hydroxide (NaOH) to reduce any inconsistency in pH level caused by interference; however, further process optimization (in terms of NaOH amount or centrifugation/filtration techniques) is required to prevent denaturing of the biomolecules. The LOD and sensitivity of this sensor were reported to be 0.03 μM and 9044.6 µA mM^−1^ cm^−2^, respectively within a 0.1–1000 µM linear working range. 

As the device sensitivity highly depends on various structural factors such as shape, size, active site density, porosity, etc., currently researchers have been focusing on sensor structures with the aim of improving overall performance [59]. Recently, a unique non-enzymatic sensor was developed by Coyle and group by combining a gold (Au) honeycomb-like framework anchored with a Co_3_O_4_ sharp needle, where the needle coating on a porous scaffold provides excellent electrochemical sensing. The detection mechanism involves sets of oxidation reactions with glucose at the electrode surface. The device could exhibit varying sensitivity from 2.014 mA mM^−1^ cm^−2^ to 0.011 mA mM^−1^ cm^−2^ within a 0–10 mM glucose range, with an LOD as low as 20 μM [60]. This sensor can detect glucose selectively in the presence of interfering species such as ascorbic acid, uric acid, cortisol and dopamine, making it suitable for real-life diagnostic applications. 

The above section discussed technology advancements made while exploiting the convenience provided by saliva for glucose sensing to date. Overall, there has been a drastic shift observed from optical measuring systems, which involve complex pre-processing with expensive reagents and sophisticated instruments, to microchips and microfluidic devices for at-home determination of salivary glucose levels [61]. Despite all of these improvements, a shortcoming pertaining to lower glucose concentrations in saliva still prevails. Hence, researchers are still working on perfecting the sensors’ performance in terms of detection technology, device structure, sensitivity, detection limit and selectivity, etc., in order to modify and enhance existing salivary sensors for continuous monitoring of glucose. The performance of various salivary glucose sensors is summarized in Table 1.

### 2.2. Tear-Based Glucose Sensors

Tears are produced by the lachrymal gland situated within the circumference above the lateral end of the eye. They assist in an anti-fouling mechanism and form a protective film for the eye [66]. Compared to other biological fluids, tears are superior in terms of being a target for wearable devices, as biomarkers present in tears diffuse directly from the blood, and their glucose concentrations are more closely correlated [67]. The average glucose concentration in tear samples from healthy subjects is about 3.6 mg/dL, whereas in diabetic patients, it is at 16.6 mg/dL. [68] One of the earliest works on glucose wearable enzymatic sensors using tears was carried out by Kudo et al., based on electrochemical principles of functional polymers using Soft-MEMS techniques [69]. The device could detect the glucose level within a 0.06–2.00 mmol/l range, with a 0.997 correlation coefficient with blood glucose. Since this research, various types of tear-based glucose sensors have been developed in the past decades using different advanced techniques to improve device performance. 

Peng and co-workers developed an enzymatic coulometry electrode that measures the change in analyte concentration during electrolysis through current measurement for glucose detection [70]. The inner layer of electrode is composed of Pt/Ir wire, where glucose oxidase is immobilized by utilizing excess oxygen, whereas the outer layer of Nafion and 1,3-diaminobenzene acts as mediator for electroactive interference, resulting in enhanced selectivity. This device is capable of detecting glucose in tears in amounts as low as 3 μL within a 2–20 mM detection range and exhibits higher selectivity with error percentages of 2.1%, 5.8%, and 2.5%, respectively, for ascorbic acid (at 100 μM), uric acid (at 100 μM), and acetaminophen (at 10 μM). The sensor was directly tested by measuring the glucose concentration in a tear sample collected from the conjunctiva under the lower eyelid of an anesthetized rabbit; however, the sample collection process was complicated. In these electrochemical platforms, the current output is correlated to the glucose concentration during the measurement.

Owing to the difficulties in sample collection, a prototype of a microfluidics-based self-monitoring, non-invasive enzymatic device was proposed by Belle et al. The device was projected to have a soft polyurethane foam instead of the traditional glass capillary tubes for tear collection and a micromechanical pump to transfer the collected sample to the sensing well containing the necessary enzymes and reactants for electrochemical detection. Within 10 seconds, this sensor can detect glucose concentrations in tear samples ranging from 0.72 mg dL^−1^ to 111.6 mg dL^−1^, which can be extrapolated to 20 mg dL^−1^ to 600 mg dL^−1^ in blood glucose. The device was named “TOUCH”, reflecting its sensing capability with a gentle touch to the eye [71].

**Figure 5:**
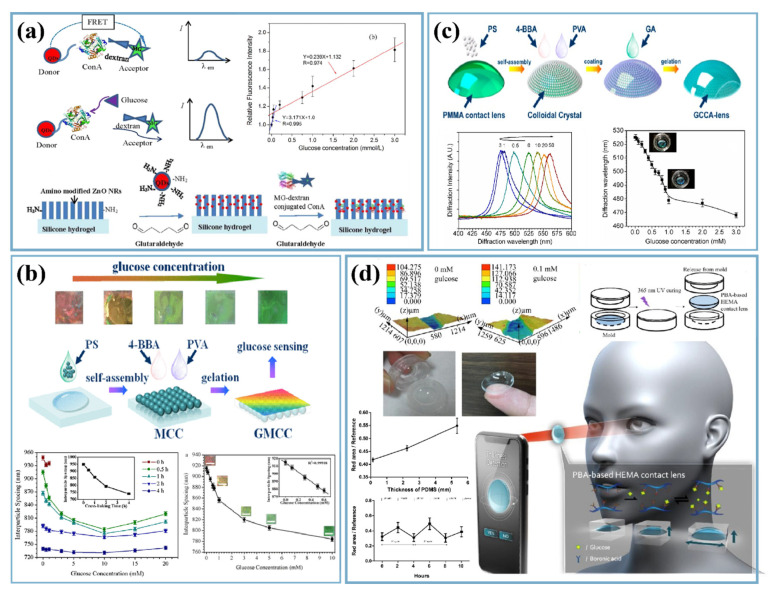
(**a**) Working principle behind designed FRET transducer in detecting glucose. (Reproduced with permission from Chen et al., Biosens. Bioelectron., vol. 91, pp. 393–399, 2016 [72]. Copyright 2016, Elsevier Ltd.) (**b**) Construction of gelated monolayered colloidal crystal (GMCC) 2D structure and its color correlation with glucose content. (Reproduced with permission from Chen et al., 2018 [74]. Copyright 2018, American Chemical Society.) (**c**) The process for preparation of the 4-BBA-PVA GCCA-lens. (Reproduced with permission from Ruan et al., Polymers, vol. 9, no. 125, pp. 1–12, 2017 [76]. Copyright 2017, authors under open access licenses.) (**d**) Portable non-invasive contact lens with an imaging program in a smartphone and the mechanism involved. (Reproduced with permission from Lin et al., Sensors, vol. 18, no. 10, Sep. 2018 [77]. Copyright 2018, authors under open access licenses).

In terms of scalability and cost-effectiveness, FRET is an inexpensive method of enzymatic glucose detection, as illustrated in Figure 5a. This is a distance-dependent nonradioactive energy transfer process from a fluorophore donor to an acceptor. Here, cadmium selenium core zinc sulfide shell (CdSe/ZnS) quantum dots were used as donors and malachite green dextran as acceptor conjugated to concanavalin A enzyme, which has specific glucose affinity. The sensor was immobilized onto ZnO nanorods, creating patterns that enhance output signal intensity and enable easy identification under fluorescence microscopy. For practical applications, contact lenses were developed based on this technology with patterned nanostructured FRET sensors deposited on silicon hydrogels. Here, a USB-powered portable digital microscope was used to capture and measure the fluorescence intensity of the glucose sensor conjugated onto ZnO nanorods. The device was tested on animal subjects, and it showed linearity within a 0.03–3 mmol/L glucose concentration range, covering both diabetic (>0.35 ± 0.04 mmol/L) and healthy people (0.16 ± 0.03 mmol/L) [72]. 

In regard to the property of biocompatibility, another nanostructured catalyst-based enzymatic colorimetric glucose sensor was prepared with peroxidase-mimicking properties. Recently, Xiong et al. developed an enzymatic electrochemical sensor by synthesizing porous Co_3_O_4_ hollow nano polyhedrons as functionalized gate electrodes on a ZIF-67 template in solution-gated graphene transistors (SGGT) for tear glucose and uric acid detection. The formation of H_2_O_2_ due to glucose oxidation at the gate and direct electron oxidation were the sensing mechanisms involved, respectively, for both glucose and uric acid. The device quantitatively detected glucose concentrations in real tear samples at 323.2 ± 16.1 μM with a 10 nM LOD. The device sensitivity was further modified by immobilization of GOx-CHIT on the Co_3_O_4_/Au gate electrodes, which improved the LOD to 100 nM [73]. To overcome limitations such as eye irritation, blurred vision, and damage to the inner eyes, Park et al. fabricated a contact lens that was transparent, soft, and stretchable with wireless technology. The contact lens is based on a hybrid substrate of photocurable optical polymer that is photolithographically patterned on a Cu sacrificial layer of 800 nm thickness and coated with a silicone elastomeric layer. Wireless operations were carried out by the power transfer circuit of an inductive antenna and rectifier with stretchable and transparent electrodes that consisted of AgNFs. This device showed long-term stability for up to 48 h with sensitivity of ~ 22.72% mM^−1^ and an LOD of 12.57 μM [29].

Recently, photonic crystal (PC) materials have drawn huge attention as non-invasive tear-based enzymatic glucose sensors. These sensors are capable of detecting glucose levels in tears through the efficient diffraction of visible light, which exhibits structural color changes in the presence of glucose. Chen et al. developed a photonic crystal material with a high-order monolayered colloidal crystal of polystyrene (PS) (Figure 5b). This 2D structure was then coated with 4-boronobenzaldehyde (4-BBA) functionalized poly(vinyl alcohol) (PVA) hydrogel, which changes color from red to green through yellow over the range of glucose levels from 0 to 20 mM within 180 s [74]. It has been shown that different photonic crystals such as naturally occurring opaline colloidal photonic crystals (CPCs), inverted opaline 3D CPCs, 2D CPCs, and hydrogel-based 2D and 3D CPCs are excellent candidates for sensing purposes in the detection of glucose using the same principle of diffraction [75].

Currently, the multifluid detection technique is becoming popular, as it facilitates glucose detection in various fluids using the same device. This supports the idea of utilizing an alternative biological fluid when a given fluid of choice is unavailable or in short supply. In this regard, Beigi and co-workers have developed an enzymatic tear as well as saliva sensor using a cobalt hydroxide/porous graphene nanocomposite ((PG/Co(OH)_2_) based multifluidic device that shows excellent catalytic activity towards luminol as a chemiluminescent active substrate. It is equipped with a homemade power supply (Potentiostat/Galvanostat, Model: NCF-PGS 2012, Tehran, Iran). The H_2_O_2_ produced from the oxidation of glucose helps luminol attach to the nanocomposite, which is detected through chemiluminescence signal intensities. This device showed good sensitivity with the ability to detect glucose levels in the range of 3.0 × 10^−9^ to 4.0 × 10^−5^ M and an LOD of 6.4 × 10^−10^ M [65].

Similarly, Vinita et al. developed a peroxidase mimetic enzymatic nanoporous coordination polymer (NPCP). Here, 4-Amino-3-hydrazino-5-mercapto-1,2,4-triazole (AHMT) and palladium salt (PdCl_2_) were used as precursors to synthesize AHMT-Pd NPCP, which acts as a catalyst during the oxidation of TMB by H_2_O_2_ using a hydrothermal process. Both the AHMT-Pd NPCP catalyst and TMB were added in equal amounts to the tear and saliva test samples to record the absorbance spectra at 652 nm for glucose level monitoring. Besides the absorption spectra, a preliminary visual analysis can be made by studying the color change from yellowish green (0–8 μM) to bluish green (8–12 μM) for diabetic patients. This device showed good selectivity, repeatability and reproducibility with a working range of 1–300 μM and an LOD of 0.061 μM for tear and 0.091 μM for saliva [63]. Kownacka et al. developed an enzymatic prototype device named the NovioSense Glucose Sensor (Nijmegen, Netherlands). This device can be worn under the lower eye lid for continuous monitoring of glucose in tear fluid. Here, all three electrodes (reference, counter and working) were made from uncoated Pt/Ir wires. Glucose level quantification was achieved through chronoamperometric measurement when H_2_O_2_ was detected on the working electrode during enzymatic reaction [28].

Like the salivary sensor, demand for non-enzymatic ocular glucose sensors has also been rising day by day. Sha and group synthesized reduced graphene oxide (GO)-niobium pentoxide Nb_2_O_5_ nanoparticle composites using hydrothermal synthesis for tear glucose detection. The sensing properties of this composite were imparted by the forward-biased nano Schottky interfacial barrier between reduced GO and Nb_2_O_5_. This non-enzymatic electrochemical sensor uses Pt wire as the counter electrode and Ag|AgCl as the reference electrode, which converts glucose to gluconolactone by transferring electrons from the conduction band to Fermi energy level. The device achieves 3.23 µA mM^-1^ sensitivity within a 1 mM to 10 mM linear detection range [62]. A nonenzymatic tear-based glucose detection technique for in situ glucose monitoring through a lens was achieved by attaching a gelated colloidal crystal array (GCCA) to it [76]. The composition of the sensor involves 3D polystyrene (PS) crystalline colloidal arrays embedded within a 4-boronobenzaldehyde (4-BBA)-modified poly(vinyl alcohol) (PVA) hydrogel matrix and attached to a rigid gas-permeable contact lens, polymerized from polymethyl methacrylate (PMMA) (Figure 5c). Owing to its dielectric periodicity, the GCCA is able to diffract visible light within the 567–468 nm electromagnetic spectrum range upon interaction with glucose, which changes the lens’ color. The detection mechanism involves change in spaces between PS particles when borate ions and diols within the hydrogel matrix react with glucose. The lenses can work within the detection range of 0–50 mM with an LOD of 0.05 mM [76].

Another type of contact lens, developed by Lin et al., does not use any power source or spectrometers for glucose detection, unlike others. These contact lenses were developed using phenyl boronic acid-based hydroxyethyl methacrylate hydrogel, which can generate differential osmotic pressure and is able to reversibly swell and shrink with varying glucose concentrations. Upon exposure of the tear glucose to the lens, a simple image is captured on a smartphone as illustrated in Figure 5d. Images taken by smartphones with photosensitive sensors in the presence of red-light emitting diodes were processed via MATLAB to find the red-light area and then correlate it to the glucose concentration [77]. 

Even though tears are a great choice for glucose level monitoring, sample collection difficulties due to small sample volume, ease of evaporation, and variations in production among individuals for in vivo methods that use reflex tears generated during emotional or mechanical stimulation have seriously hindered real-life applications of ocular glucose sensors. Although a few of these challenges have been addressed with in vitro sample collection methods using basal tears that provide stable blood-related composition, there is still a need for improvement in terms of sensitivity, selectivity, response time, LOD, etc. The performance of various ocular glucose sensors is summarized in Table 2.

### 2.3. Urine-Based Glucose Sensors

Urine is a liquid by-product of human metabolism that contains many analytes such as urea, uric acid, and creatinine, facilitating a platform to monitor overall health status. Typically, for people under normal conditions, the urine samples do not show any existence of glucose. However, for diabetic patients, when the glucose content is between 0 mg/dL and 15 mg/dL (known as renal threshold of glucose), it can be detected and quantified in urine [79].

Researchers have made great progress in the area of urinary sensors by developing various techniques and studying the properties of change in urinary glucose levels in correlation with blood glucose concentration. In this line of research, photonic crystals that are dielectric structures with a certain band gap and allow light propagation for a particular frequency range have gained considerable attention in the past few years [80]. In these kind of devices, light flows across slotted structures that help in achieving high sensitivity by confining spatial [81], temporal [82] and optical [83] mode peaks within the analyte.

One of the detection techniques in photonic crystals involves calculating absorbance energy in the photonic slots using the Maxwell equation E(t) = (Eo − Er) e−(αt+ β d) where E(t), Eo and Er are associated with transmitted, incident and reflected energies, respectively, t is the glass thickness, and α and β are absorption coefficients for glass and urine, respectively. Based on this principle, Swain et al. developed a 2D triangular photonic structure using glass (VITRON-IG) as background material. The experimental setup comprised 2D crystals holding the urine samples, a light source, a photo detector for detecting the energy of the transmitted waves, and an LCD-interfaced Arduino board for displaying the results. Here, the 2D crystal is set on a glass background with a lattice constant of 1 μm, and an incident beam of wavelength 1550 nm, which facilitates detecting glucose concentrations in the range of 0–10 gm/dL corresponding to the output energy increase from 0.719 eV to 0.722 eV [84].

**Figure 6:**
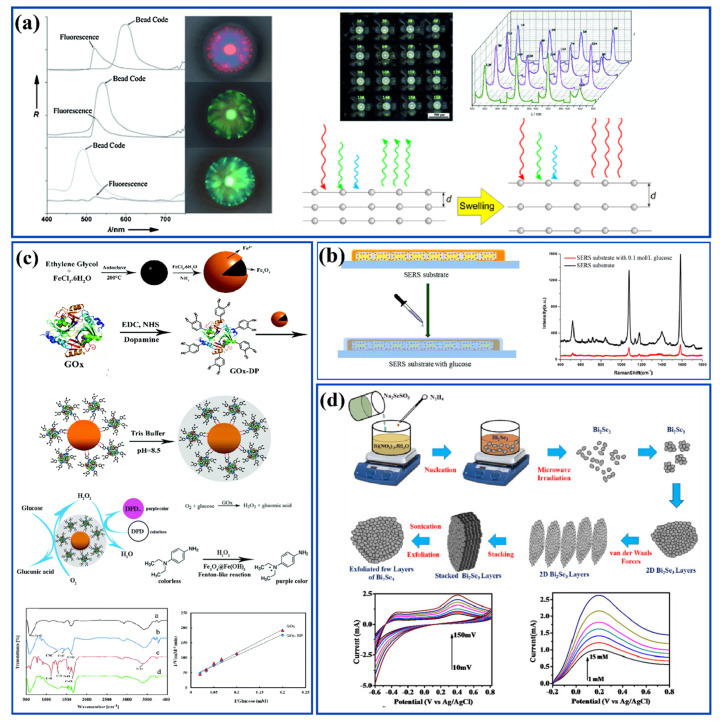
(**a**) Digital image of a 4×4 photonic crystal sensor array; reflection spectra of the beads in the 4×4 array; fluorescence and optical reflection spectra; physical and chemical sensing mechanism. (Reproduced with permission from Fenzl et al., Angew. Chem. Int. Ed., vol. 53, no. 13, pp. 3318–3335, 2014 [85]. Copyright 2014, John Wiley and Sons.) (**b**) Schematic representation of the probe preparation and testing of glucose. (Reproduced with permission from Zhu et al., J. Mater. Chem. C, pp. 1–29, 2019 [82]. Copyright 2019, Royal Society of Chemistry.) (**c**) Peroxidase-like activity of Fe3O4–Fe(OH)3@GOx–polyDA as a biosensing platform for colorimetric detection of H_2_O_2_ and glucose. Color change representation of DPD before and after the catalytic reaction. (Reproduced with permission from Pezhhan et al., R. Soc. Chem., vol. Analytical, pp. 1–13, 2019 [94]. Copyright 2019, Royal Society of Chemistry.) (**d**) Schematic illustration of the formation of stacked layers of Bi2Se3 and exfoliation process. (Reproduced with permission from Savariraj et al., J. Electroanal. Chem., p. 113629, 2019 [99]. Copyright 2019, Elsevier Ltd.).

Following this, an advancement in 2D photonic crystals with a hexagonal lattice was reported, which was developed on the basis of the principle of the resonance cavity (Figure 6a). Here, once the urine sample is placed inside a particular cavity, the primary resonance in the sensor structure is selected by the respective resonant wavelength, providing the corresponding glucose density [85]. In this study, an analysis was performed to determine the concentration of glucose within a 1.19760504~1.19760510 µm wavelength range, resulting in a 0.2 × 10^−7^ RIU (wavelength resolution) and sensitivity ranging between 1250–10,000 nm/RIU [85].

In another study, K. Aidinis et al. developed two variants of 2D hexagonal photonic lattice crystal-based bio-optical sensors using an identical shape and structure, where sensitivity was modulated by varying the size and quality factor (QF) of the sensor [83]. In these crystals, the glucose level was measured by detecting change in the refractive index of the urine samples and correlating the respective resonance frequencies with glucose concentration levels. The sensitivity reported for the sensor with a size of 14.93  ×  8.8 μm^2^ was 500 nm/RIU with 3800 QF, while for the 15.7  ×  9.47  μm^2^ sensor size, it was 500  nm / RIU with 5540 QF. Although an extraordinary number of challenges can be solved using photonic crystals, there are a few major drawbacks, such as high fabrication cost, the need for pre-sample treatment, and biofouling of delicate crystal structures in the absence of a clean room environment [86].

To avoid these issues, research has focused on various other techniques for glucose detection, one of which is surface plasmon resonance (SPR). SPR takes place when an electrically conductive surface is stroked by polarized light at the interface of two media, resulting in the generation of electron charge density waves called plasmons, reducing the intensity of reflected light at a specific angle in proportion to the mass on a sensor surface [87]. Recently, Yuan and co-workers developed a fiber-optic SPR-based glucose sensor using p-mercapto phenyl boronic acid (PMBA) modified Au nanoparticles for detecting different types of saccharides such as fructose and galactose along with glucose [81]. Although the sensor was highly sensitive towards all saccharides, it lacked selectivity towards glucose. To address this issue, Au nanoparticles (Au NPs) modified with 2-aminoethanethiol (AET) and PMBA were employed. In the presence of these nanoparticles, glucose binds with PMBA on the optical fibers and forms secondary binding with the diols, leading to the formation of a sandwich structure depending on the glucose concentration. The device exhibits an LOD of 80 nM within the detection range of 0.01–30 mM, which is evidently lower than the physiological blood glucose level [81].

Another method utilizes surface enhanced Raman spectroscopy (SERS), which is a spectroscopic technique that simultaneously combines vibrational spectroscopies with sensitivity down to single molecules for the enhancement provided by plasmonic effects and uses a distinct Raman peak to detect glucose [88]. In this regard, Chen and co-workers synthesized silver-coated gold nanorods (Au@AgNR) for quantitative analysis of glucose content within urine [89]. In this sensor, 4-Mercaptophenyl-boronic acid (4-MPBA) and 4-Cyanophenylboronic acid (4-CPBA) were used to capture glucose. The distinctive Raman peak of the Cyano group in 4-CPBA was used for glucose sensing, as the peak lies in the biologically silent region, resulting in no interference from other molecules. Not only were the SERS substrates optimized to obtain the best plasmonic activity, but they were also found to be stable for a longer duration. The LOD of the obtained sensor was found to be 10^−8^ M [89].

Using the same technique, Zhu et al. compared the performance between Ag-coated Au nano bones with bare Au nano bone devices, where the Ag coating enhanced the SERS activity by eight-fold. The SERS activity was further enhanced 4 times by decorating the nano bone devices with GO. Here, nano bones were prepared using seed-mediated growth, during which the GO was decorated on the Ag-coated Au nano bones as shown in Figure 6b. 4-Mercaptophenyl-boronic acid (4-MBA) was used as the Raman probe molecule. With higher glucose concentrations, the thickness of the layer deposited on the nano bone structure increases, resulting in low Raman signal intensity. It was noted that Raman peak intensity at 1585 cm^−1^ linearly decreased with an increase in the glucose concentration in the range of 10~107 nmol/L, with an LOD of 2.61 nmol/L [82].

Fluorescence is an alternative technique for glucose detection, where fluorophores (complex protein molecules) are used for absorbing energy of a certain wavelength while emitting energy of another. In urinary sensors that use this technique, the glucose concentration is measured by detecting the change in fluorescence of the fluorophores [90]. Recently, Liang et al. synthesized boron-doped carbon nanoparticles (NB-CNPs) with pH-sensitive fluorescent properties using a one-step solid-phase approach [91]. Here, a boronic acid-triggered specific reaction was utilized for the detection of glucose with an excitation wavelength of 370 nm within the range of 0–900 μM. The limit of detection for glucose was noted to be as low as 1.8 μM [92].

Recently, enzymes have been extensively explored for their ability to aid in detecting glucose; hence, research on enzymatic based glucose sensors is being continued. A reusable Pt electrode for glucose detection in urine with high selectivity was developed by Go et al. [93]. The electrode evidently provided stable measurements in up to 200 tests with a simple water rinse; this stability was attributed to the solid structure of the ascorbic oxidase and glucose oxidase composite coated over the electrode. This multi-layered enzymatic composite coating helped in increasing the number of electrons available to the electrode from the glucose oxidase layer without interfering with the ascorbic acid in the urine. The detection capability of these electrodes was reported to be between 0 mg/dL and 1000 mg/dL [93].

Another study involved the development of a selective and reusable probe by Pezhhan et al. based on the encapsulation of GOx to immobilize the enzyme [94]. Using the hydrothermal method, nanoparticles with Fe(OH)_3_ shells and Fe_3_O_4_ core were developed and used in connecting dopamine (DA), which acts as an anchor, with the nanoparticles and GOx. The DA grafted with GOx was further mixed with Fe(OH)_3_@Fe_3_O_4_ to form Fe_3_O_4_-Fe(OH)_3_@DA-GOx. This DA-GOx was polymerized through cross-linking for better stability to obtain Fe_3_O_4_–Fe(OH)_3_@polyDA-GOx, shown in Figure 6c The colorimetric application of this artificial enzyme comes from the cascading reaction, wherein first the glucose is oxidized to gluconic acid in the presence of oxygen and produces H_2_O_2_, which then undergoes oxidation through N,N-diethyl-p-phenylenediamine sulfate (DPD) substrate to impart color, facilitating glucose detection. This sensor showed a limit of detection of up to 3 μM [94].

Nanozymes (enzyme-mimicking nanoparticles) have recently gained attention for their use in biosensing applications. These artificial enzymes are known for their ability to sustain a wide range of temperatures and pH values. Among their advantages, low fabrication costs and catalytic activity in various enzymatic reactions rank at the top. Colloidal solutions of nanozymes have been reportedly used for several glucose sensing applications, although their use directly in the biological fluids is not so common. Karim et al. developed free-standing nanozymes loaded on cotton fabric for rapid glucose estimation [95]. For this, Ag+ nanoparticles were deposited on individual threads of a 3D woven cotton matrix through the process of electroless metal deposition. The cotton fabric was sensitized, and then Pd nanoparticles were deposited. Further, the fabric was exposed to diamine silver complex in the presence of a reducing agent, glucose. Quantification of deposited glucose was achieved using atomic emission spectroscopy. A linear dynamic operating range potential of 0.1–2 mM was obtained.

Yang et al. reported the development of a paper-based glucose sensor using a bimetallic reduced graphene electrode with enzyme-mimicking activity [96]. Iron-palladium(Fe-Pd) nanoparticles were utilized here. When the electrode encountered the glucose samples with different concentrations of up to 10 mg/mL of GOx, the obtained absorbance was recorded and compared with the calibration curves for glucose concentrations. The limit of detection was calculated to be 1.76 μm. Another paper-based non-invasive urinary glucose sensor with (GOx/PEDOT:PSS) sensing electrodes involved a sensing strip, amplifier circuit and a visual readout. Here, the strip comprised five enzyme-based activated electrodes, with each connected to a particular indicator circuit that triggered an LED when a predefined glucose concentration was reached. It was reported that the sensor could generate visual responses in less than 2 min during glucose concentration detection in urine. The device was capable of detecting five glucose concentrations discretely ranged from 1 to 5 mM, with 1.35 μA/mM sensitivity [27]. Another study involved fabrication of a laminate-structured portable urine glucose sensor consisting of four consecutive layers with selective functionalities. Here, Pt was used for the working and counter electrodes, and a thin film of Ag/AgCl composite was utilized for the reference electrode. The reference electrode acted as potential stabilizer after device immersion within the urine solution. A fluorinated polymer coating used for the outermost protective layer enhanced device longevity by 1 year. This sensor showed a measurement range within 10–2000 mg/dL [26].

Apart from enzymatic urinary sensors, non-enzymatic sensors are being developed to address the drawbacks discussed earlier, and can directly oxidize glucose in the sample without the need for an enzyme [97]. Recently, Sun et al. developed a non-enzymatic electrochemical glucose sensor containing copper (Cu) -based metal–organic framework (Cu-MOF) modified electrodes with anti-interference properties [98]. They achieved a detection limit of 10.5 nM and sensitivity of 89 μA mM^−1^ cm^−2^ with selective oxidation of glucose. The amperometric response of Cu-MOF-modified GC electrode to glucose at 0.5 V in 0.01 M NaOH was found to be a linear relation. The mechanism for this non-enzymatic electrocatalytic activity involves oxidation of Cu in Cu(II)-MOF as well as oxidation of glucose to gluconate. Another non-enzymatic electrochemical biosensor with cobalt–iron (CoFe) coated N-graphene electrodes was developed. Platinum was used as the auxiliary electrode and Ag/AgCl as the reference electrode in the construction of this biosensor. Cyclic voltammetry and amperometry measurements were used for glucose detection with a DC bias of +9 V between the working electrode and Pt electrode. Sensitivity of 476.67 µA cm^−2^ mM^−1^ with an LOD of 37.7 μM was obtained. The detection mechanism involved pre monolayer formation of hydroxyl group by adsorption onto active sites followed by oxidation of cobalt peroxide (CoO_2_) and oxidation of glucose to gluconolactone [28]. In the same field of research, 3D bismuth selenide (Bi_2_Se_3_) nanostructures were synthesized by A.D. Savariraj and group (Figure 6d) [99]. Bi_2_Se_3_ layers played the key role in increasing the electron transfer between glucose and the electrode. The sensor has reported sensitivity of 0.112 μA μM^−1^ in a 10–100 μM linear range of detection with a detection limit of 6.1 µM.

Urinalysis has been the prevailing method for detection and management of disorders such as urinary tract infections and kidney disease for decades. Recently, it has been extended to diabetes which possess great potential in continuous monitoring of glucose. Further research and innovation in urinary glucose sensors for achieving higher sensitivity along with selectivity are still being pursued while overcoming the limitations incurred, which are detailed in the conclusion. The performance of various urinary glucose sensors is summarized in Table 3.

### 2.4. Sweat- and Interstitial Fluid-Based Glucose Sensors

**Figure 7:**
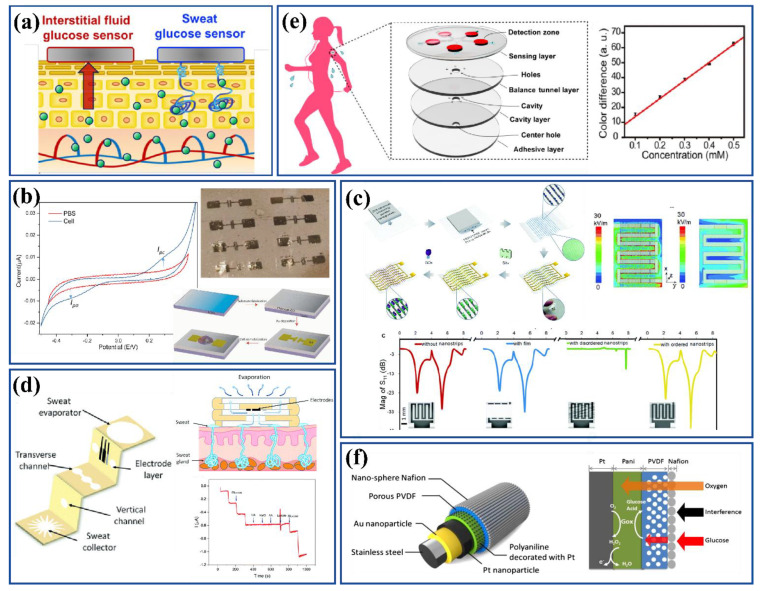
(**a**) Schematic representation of glucose monitoring sample generation. (Reproduced with permission from Kim et al., Talanta, vol. 177, pp. 163–170, 2018 [101]. Copyright 2018, Elsevier Ltd.) (**b**) Illustration of chitosan-GO substrate fabrication. (Reproduced with permission from Janyasupab et al., 2018 IEEE-EMBS Conf. Biomed. Eng. Sci. IECBES, pp. 577–582, 2018 [110]. Copyright 2018, IEEE.) (**c**) Representation of nanostrip biosensor fabrication and enzyme immobilization. (Reproduced with permission from Xui et al., Nanoscale Horiz., 2020 [96]. Copyright 2020, Royal Society of Chemistry.) (**d**) Graphics of the 3D-PMED sensor applied on human skin. (Reproduced with permission from Cao et al., R. Soc. Chem., vol. 9, pp. 5674–5681, 2019 [114]. Copyright 2019, Royal Society of Chemistry.) (**e**) Layers and zones of the glucose sensor. (Reproduced with permission from Xiao et al., Anal. Chem., 2019 [36]. Copyright 2019, American Chemical Society.) (**f**) Illustration of nanostructure layers and working principle of the sensor. (Reproduced with permission from Chen et al., Biosens. Bioelectron., vol. 74, pp. 1047–1052, 2015 [121]. Copyright 2015, Elsevier Ltd.).

Epidermal glucose sensing systems, with their capability for noninvasive continuous monitoring, offer opportunities for use in therapeutic drug delivery systems. These types of glucose sensors have been developed for both sweat and interstitial fluids. Glucose that diffuses from the blood vessels to these bio-fluids through endothelial cells or sweat glands has a direct correlation with blood glucose concentration, reflecting diabetic status (Figure 7a) [101]. The condition of diabetes mellitus is known to exist when the glucose concentration in sweat ranges from 0.1–50 mg/dL; however, in healthy subjects no value is detected [102]. Sweat is a promising biofluid for the development of sensors irrespective of issues such as varying corelative factors, low sweat production by some individuals, the need for personalization, etc. Transdermal drug delivery for controlling blood glucose levels is a solution aimed at reducing drug dosage while avoiding intestinal problems. To ensure an accurate drug delivery system, continuous glucose detection is important, and sweat plays an important role in such collaborative designs.

Mostly using noninvasive or minimally invasive techniques, various types of thin film-, tape-, tattoo- and patch-based sensors have been developed for epidermal glucose sensing owing to their flexibility, ease of construction and use, low cost, etc. Recently, Munje et al. fabricated a novel electrochemical biosensing thin film for impedance-based detection of glucose levels in human sweat [103]. The working principle was based on non-Faradaic electron-ionic charge transfer between the electrical double layers formed within the thin alternating stacked films of gold/zinc oxide embedded in a porous polyamide substrate. Non-Faradaic electrochemical impedance spectroscopy (EIS) was used to study the change in impedance with varying glucose concentrations. The device showed sensitivity of 14.5 μA mM^−1^ cm^−2^ with the ability to correlate the quantity of glucose selectivity in the range of 0.01–200 mg/dL exhibiting an LOD of 0.1 mg/dL. Another flexible thin-film electrochemical sensor was developed by Wang et al. using gold-plated PET substrate acting as working electrode [104]. The device showed the ability to detect glucose in purified sweat samples with a linear range of 0.02–1.11 mM and a 2.7 μM LOD. This sensor showed selectivity of 22.05 μA mM^−1^ cm^−2^ against glucose in the presence of ascorbic acid, urea, dopamine, uric acid and lactic acid.

In the same field of research, wearable adhesive tapes are an ideal sweat-based enzymatic biosensor, as they can be attached directly on skin, forming close contact with the epidermis, and are capable of detecting multiple biomarkers simultaneously. Luo et al. fabricated an electrochemical sensor on adhesive tapes for detection of glucose and lactate in sweat [105]. The sensor was prepared by heating and printing carbon graphite ink on adhesive tape to develop working as well as counter electrodes with Ag/AgCl as reference electrode. The working electrode was further deposited with a thin layer of Prussian blue [106] to enhance the electron transfer and selectivity towards H_2_O_2_, followed by hydrophilic modifications, then casting of enzymes, chitosan and CNT. Here, the interaction of Prussian blue with H_2_O_2_ and immobilization of lactate oxidase or glucose oxidase on the working electrode control output current during detection. The device was reported to have a detection time of 5 min with a linear range of 2.38 mM to 14.29 mM and an LOD of 3.84 μM [105].

Another study involved the use of PEDOT:PSS spray coated on laser-induced graphene (LIG) flakes to develop a biosensor for glucose and pH sensing in sweat. While the robustness of the prepared electrode was improved by utilizing porous LIG, the electrochemical activity was enhanced by the electrodeposition of platinum and palladium nanoparticles. The working mechanism involved electrocatalytic oxidation of glucose onto the electrode. A wide linear range of 10 µM–9.2 mM and LOD of 3 µM were reported along with high sensitivity during amperometric response [107]. In another study, a ratiometric electrochemical sensor was fabricated by Wang et al. with a Schiff-base polymer (SBPthi) 2D mesoporous nanosheet firmly adhered onto a glass carbon electrode. Here, p-benzaldehyde-thionine amine-aldehyde was polymerized via condensation reaction to obtain the 2D nanosheet, where the pores were used to load GOx. During cyclic voltammetry measurements, two pairs of redox peaks were obtained at −0.05 V and −0.2 V due to the oxidation of SBPthi. The ratio of the SBPthi peak to oxygen reduction peak was used to determine the glucose content, as the two peaks used for reference were far apart from the oxygen peak, avoiding any overlap and increasing the window for glucose content that could be detected. Linear ranges of 0.82 μM–4.0 mM and 1.97 μM–4.0 mM and LODs of 0.27 μM and 0.66 μM, respectively, were reported when the reference peaks were −0.05 V and −0.2 V [108].

Apart from just glucose level monitoring, Kafi and coworkers fabricated a chitosan-graphene oxide (CS-GO) based array of ultra-thin biosensors for diabetic wound monitoring as well. Here, human dermal fibroblast (HDF) cells were immobilized on a graphene oxide-crosslinked chitosan substrate surface, which was utilized for cell health monitoring. The device was further engineered by depositing an array of micro-gap Au electrodes for glucose detection along with monitoring of the cell health proliferation rate at the wound site. In this sensor, arginyl-glycyl-aspartic acid (RGD) peptides were used as binding motifs for the firm adhesion of CS-GO film on Au electrodes through cystine-thiol gold bonding at one end and RGD–integrin interaction with the cell surface on the other end. Glucose quantification was performed using linear swipe voltammetry. Substrate fabrication is illustrated in Figure 7b [109]. A linear range of 1 μM to 20 mM and sensitivity of 0.17 μA/mM was obtained with this sensor patch [110].

Recently, microwave signal-based implantable glucose sensors have become popular for use as highly responsive, passive and long-distance communication wireless sensors for glucose monitoring. Xue et al. synthesized ordered nanowires of PEDOT: PSS using nanoscale soft printing on a PET substrate with porous PDMS as base. This flexible setup was then doped with GOx to act as a glucose sensor. It is illustrated in Figure 7c. Integration with a nano strip antenna was proposed in this study to enhance the microwave signals, in turn improving the sensitivity of the sensor. A linear response for glucose concentration was recorded in the range 0.1 nM to 10 mM with sensitivity of 0.026 dB/log (nM) [111]. Dautta et al. developed a wireless stretchable and scalable phenylboronic acid-based biosensor for glucose detection in sweat with a hydrogel-interlayer radiofrequency (RF) resonator. Here, phenylboronic acid-hydrogels exhibited volumetric and dielectric variations in response to the varying glucose levels, which were converted to the corresponding shifts in the resonant response of the interlayer-RF sensor. The LOD was reported as 10 mg/dL with a stability period of 45 days at room temperature [112]

For ease of collection and storage of sweat samples, recently microfluidic devices have received huge attention in the field of wearable glucose sensors. Nyein and coworkers have developed a wearable microfluidic based patch using a roll-to-roll laser cutting process to electrochemically detect glucose, sodium and potassium content in sweat along with the sweating rate. This device consists of two layers, one containing the electrodes for sweat analysis and the other for collection and storage with the help of a spiral microfluidic flow channel. For transdermal drug delivery, the iontophoresis process was used, where a voltage gradient on the skin was created to study the correlation between sweat glucose and blood glucose. The device exhibited sensitivity of 1.0 nA/μM with a linear response in the 50–200 μM concentration range. It was further concluded that iontophoresis is not an accurate method for glucose sensing, as it dilutes the original glucose concentration by increasing the sweating rate [113].

In another study, a 3D paper-based microfluidic electrochemical integrated device (3D-PMED) was developed with the ability to detect sweat glucose without direct physical contact with the skin. This paper-based device was prepared on cellulose paper by modified wax screen printing to create hydrophobic boundaries. The preprinted paper was folded 5 times to create a 3D, five-layered structure where each layer played a specific role such as sweat collector, vertical channel, transverse channel, electrode containing layer and sweat evaporator. Here, sweat is transported through capillary action from the skin to the evaporator layer, which ensures continual renewal of the sweat sample to the electrodes while avoiding accumulation. All of the electrodes (carbon graphite for working and counter electrodes and Ag/AgCl for reference electrode) were also prepared by the screen printing method and loaded onto the electrode layer. A schematic diagram of the 3D-PMED applied to human skin is presented in Figure 7d. A linear detection range of 0–1.9 mM and 5 μM LOD were obtained. A real-time analysis was conducted using red ink on 3D-PMED to confirm and model the fluid flow [114].

Following a similar pattern, a five-layered microfluidic chip was developed with five microfluidic channels to conduct parallel measurements simultaneously, as shown in Figure 7e. The device was designed with five microchannels for improved reproducibility and precision, drawn outwards to a microchamber, each with a check valve to prevent backflow. Higher sensitivity towards glucose in sweat was obtained by using glucose oxidase (GOD)−peroxidase−o-dianisidine reagent, which was embedded in the microchamber of the fluidic device. A linear range of 0.1–0.5 mM with an LOD of 0.03 mM was obtained for this colorimetric biosensor [36]. A different microfluidic patch developed by Koh and group was soft, wearable and able to capture, store, and perform colorimetric sensing of sweat. PDMS layers of 500 μm and 200 μm thickness were used as bottom and top layers of the patches, respectively. A mixture of GOx, HRP, trehalose, and potassium iodide in sodium citrate buffer solution was used as a chromogenic reagent for glucose detection. Glucose levels were analyzed by monitoring changes in color from yellow iodide to brown iodine during enzymatic reaction. The device reported glucose concentrations of up to 0.1 mM with an LOD of 200 mM [30].

Implantable microneedle-based biosensing platforms have recently gained considerable attention in subcutaneous glucose sensing applications [115]. The most important factors in such applications are the biocompatibility of the device along with maintaining electrochemical activity for longer durations with higher selectivity, wider linear detection windows, and faster response times [116]. For these types of sensors, electrodes are coated with biocompatible polymers such as Nafion and polyurethane (PU). PU is one of the most versatile polymers used for developing implantable sensors due to its ease of structural and chemical modification, which can easily fine-tune the selectivity and sensitivity of the developed product. Suzana et al. used a porous layer coating composed of dexamethasone (dex)-loaded PU as mediator at the tissue–sensor interface within an implantable Medtronic MiniMed SOF-SENSORTM glucose sensor to enhance its sensitivity [117]. This coating, with 81 μm thickness and 85% porosity (average pore size 76 μm), was reported to reduce inflammation and enhance vascularization of the surrounding tissues of implants. It also increased the functional life and sensitivity of the implantable glucose sensors over a 21-day time period compared to both bare and dex-free porous controls. In the same line of research, Wang and team fabricated coaxial polyurethane gelatin electrospun membranes with a mean fiber diameter ranging from 261 nm to 1328 nm by optimizing various process parameters (such as applied voltage, working distance, flow rate, etc.) and solution concentrations to obtain desirable biochemical activity along with targeted mechanical properties and selectivity. The thin gelatin shell (34%) with thicker coaxial fiber membrane (mean fiber diameter, ~1133 nm) was reported to maintain the sensitivity and linearity of the sensor, with the longest storage period of 84 days. The study provided a pathway to coatings as a pioneer of such combinations on glucose sensing electrodes, inspiring innovation in a similar direction [118]. Another study by Ribet et al. involved the development of an enzymatic microneedle-based ISF sensor that can act as a CGM device when inserted within the dermal layer of skin. Here, the device was fabricated by integrating an electrochemical sensing probe into a hollow microneedle using standard silicon microfabrication technologies. Platinum was used for both the counter and working electrodes, whereas for pseudo-reference electrodes, iridium oxide (IrOx) was used. For in vitro study, this microneedle-based sensor showed stability for up to 4 days with sensitivity of 1.5 nA/mM and linearity of up to 14 mM [33].

Poulos et al. developed a silane precursor-based xerogel-layered biosensor by mixing propyl- trimethoxysilane, octyl-trimethoxysilane, isobutyl-trimethoxysilane, and hydroxymethyl-trimethoxysilane. The xerogel layer serves the purpose of housing the enzymatic reaction, acting as a signal transducer, and controls diffusion of both glucose and O_2_ on the enzyme-doped layer as well as eliminates redox activity of common interferents. This sensor evidently exhibited dynamic linear ranges of detection ≥24–28 mM glucose with low response times (ranging within ~ 2.5–30 s) [119]. Ribet et al. demonstrated a miniaturized microfluidic electrochemical biosensor for glucose detection in interstitial fluids. They fabricated the device using a layered composition of Nafion/PU/GOx-BSA-GA as the working electrode and iridium oxide as pseudo-reference electrode, which did not require deeper penetration than the dermal layer of the skin. The reported sensor thicknesses were 2.25 ± 1 µm (GOx-BSA-GA), 19.5 ± 4.5 µm (PU) and 1 ± 0.3 µm (Nafion), respectively, for the 1st, 2nd, and 3rd layers. The observed sensitivity was 1.51 nA/mM in the linearity range of 0–200 mg/dL [120].

A microneedle-based biosensing platform using porous PVDF/Nafion/PtNPs/PANI nanofibers coated on needle electrodes with a linear working range of 0–20 mM was fabricated through layer-on-layer deposition as illustrated in Figure 7f [121]. This biosensor exhibited fast response time (<30 s) with sensitivity of 0.23 μA/mM. A number of similar studies were performed with different materials such as Au/Au-multiwalled carbon nanotubes (MWCNTs)/poly-methylene blue (pMB)/FAD-Glucose dehydrogenase (FADGDH) electrodes (working within a linear range of 10–100 μM with LOD of 3 μM and sensitivity of 405.2 ± 24.1 µA cm^−2^ mM^−1^) [122], cyclic olefin copolymer and polypyrrole polymer needle/Au/p terthiophene carboxylic acid-GOx/Nafion (linear working range: 0.05–20 mM, LOD of 19.4 ± 0.62 μA and sensitivity of 0.22 μA/mM^−1^ cm^−2^) with wireless transmitters [123], hard-PDMS/polyimide/Au/rGO/GOx/Nafion electrodes (linear range: 0–30 mM, LOD: 0.198 μM) [124], which have been fabricated over the past few years with the aim of continuous glucose monitoring.

Apart from microfluidic devices and iontophoresis systems, hydro-gel touchpad-based sensors have also been developed recently for sweat analysis. These platforms provide the possibility of collecting human sweat at rest [125,126,127]. Along this research line, Lin et al. creatively utilized natural perspiration samples for wireless in situ electrochemical analysis using a thin hydrogel micropatch. This micropatch was augmented with a wireless enzymatic lactate sensing module and demonstrated excellent reproducibility, selectivity, and a response time of about 50 s [128].

Another hydrogel-based chemical touch sensor device was fabricated by Nagamine and group. The signal detected using this device reflected the concentration extracted in an agarose gel pad. Hence, the L-lactate concentration was also dependent on shift in perspiration rate and skin surface temperature [129]. In a different study, a touch-based fingertip blood-free glucose monitoring device by Sempionatto et.al. facilitated a painless and simple glucose self-testing protocol, leveraging the fast sweating rate on the fingertip for rapid assays of natural perspiration, without any sweat stimulation, along with personalized sweat-response-to-blood concentration translation. Using the personal parameters enabled a higher Pearson correlation coefficient of 0.95 and significantly higher accuracy reflected from an overall mean absolute relative difference of 7.79%, with 100% paired points in the A + B region of the Clarke error grid [130]. Recently, another hydrogel patch was fabricated by Lin et al. for sensing glucose from sweat using Prussian blue-doped poly(3,4-ethylenedioxythiophene nanocomposite electrodes. This device uses a chronoamperometry detection method and has a wider linear range of 6.25 μM–0.8 mM [131].

One of the drawbacks faced with sweat sensors in real-life applications is their performance susceptibility to varying atmospheric temperature, humidity and corresponding pH. In this regard, Lee et al. designed a multilayered-multistage transdermal drug delivery module to overcome such issues during glucose detection [132]. A wearable patch with disposable strip-type device was developed by drop casting of GOx-Graphene, Nafion and Glutaraldehyde on an electrodeposited Au electrode. Apart from the sensor, they also developed a microneedle composed of hyaluronic acid hydrogel for transdermal delivery of drugs such as metformin or chlorpropamide for type 2 diabetes. This transdermal delivery helped in reducing the dosage by completely forgoing the digestive tract. To increase the sensitivity in glucose detection, electrode surfaces were treated with gold nanoparticles. Additionally, phase-changing nanoparticles (PCNs) were embedded within the hydrogel matrix to achieve better control over drug delivery.

As the largest organ of the body, skin provides multiple locations for attaching sweat- and interstitial fluid-based sensors, making them the most convenient wearable sensors for glucose monitoring. With emphasis on issues of accuracy, precision and variable sweat level production, studies are still ongoing to make these sensors a go-to option for overall health monitoring. The performance of various sweat- and interstitial fluid-based glucose sensors is summarized in Table 4.

### 2.5. Breath-Based Glucose Sensors

Apart for bodily liquids, the exhaled breath from the human body can also be utilized to detect glucose content. The composition of exhaled breath can reflect any change in body metabolism [43]. The presence of different volatile components in the exhaled breath is directly correlated with the blood glucose level [134]. The most commonly used method is detection of acetone, which is formed due to ketone acids in the blood that are produced by the liver (known as ketoacidosis) during starvation [135]. It has been noted that for diabetic patients, the acetone level is higher than 1.8 parts per million volume (ppmv) in exhaled breath, whereas a 0.8 ppmv level is considered normal [136,137].

**Figure 8:**
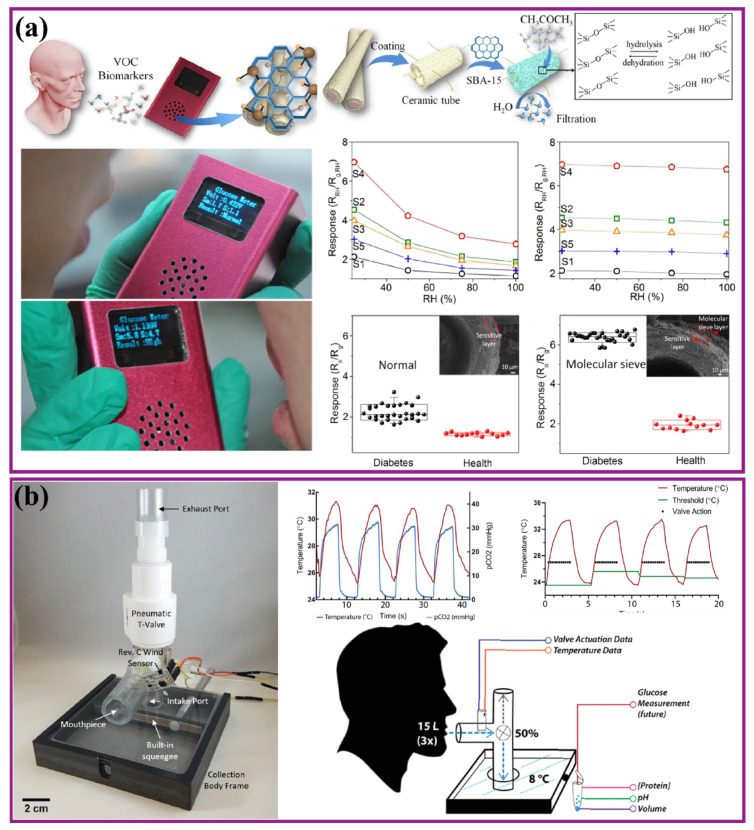
(**a**) Working principle of Pt@In2O3 core-shell NW portable sensing device along with the device response to exhaled breath from healthy as well as diabetic volunteers. (Reproduced with permission from Liu et al., NPG Asia Mater., vol. 10, no. 4, Apr. 2018 [138]. Copyright 2018, authors under open access licenses.) (**b**) Schematics of selective EBC collection device and sample collection steps involved in the EBC collection device. (Reproduced with permission from Tankasala et al., 40th Annual International Conference of the IEEE Engineering in Medicine and Biology Society (EMBC), pp. 3890–3893, 2018 [143]. Copyright 2018, IEEE).

Liu et al. synthesized a moisture-resistant acetone sensor with Pt core encapsulated in electrospun indium oxide (In_2_O_3_) nanowires (forming Pt@In_2_O_3_ core-shell nanowires) that act as sensitive layers [138]. Here, in situ breath is analyzed by keeping the device at a certain distance from the mouth, or ex situ by collecting the sample in bags with aluminum coating. Mesoporous silica was used as a filter layer due to its moisture-resistant property. In these semiconductor oxide-based gas sensors, glucose is detected by calculating the change in resistance caused by electrons released during surface chemical redox reactions between absorbed oxygen ions and target volatile gases, as illustrated in Figure 8a. The sensing range was reported to be within 10 ppb–10 ppm. Similarly, a chemical sensor system was designed by Guo et al. to collect and analyze breath samples [139]. The blood and breath samples from diabetic people were collected and corelated to draw a relation. Accuracy of up to 68.66% was obtained using this sensor. In a different study, Wang et al. developed a 3D biomimetic structurally hierarchical chemiresistor sensor that comprises a graphene sheet coating on the 3D architecture. Here, high detection selectivity of related VOCs with rapid response time ≤1 s and low LOD of 20 ppb was achieved [24].

A number of researchers have been utilizing artificial neural network (ANN) algorithms and classifiers in machine learning technique to build intelligent systems that can predict patient health upon analyzing various parameters such as acetone level, age, diet, sample timing, etc. [140,141]. Thati et al. fabricated a tin oxide (SnO_2_) breath sensor that uses ANN to obtain features from the sensor’s output waveform for acetone concentration detection [142]. This sensor has evidently detected blood glucose concentrations in a working range of 80 mg/dL to 180 mg/dL with an error limit of ±7.5 mg/dL.

Selective collection and analysis of EBC (exhaled breath condensate) from exhaled breath is another promising method for glucose detection. Tankasala et al. prepared a device that could condense more than 130 µL of EBC from 15 L of exhaled air, within 3 min [143]. Here, a portable condenser was developed to collect exhaled breath selectively through a disposable saliva-trapping mouthpiece that is connected to the intake port of the valve, as shown in Figure 8b [144]. On the other side of the mouthpiece, a temperature or wind sensor was used for exhaled breath detection. This technique requires more study, as many issues such as dead space air, performance reliability, and dependence on temperature or pH (potential of hydrogen) still persist. Another innovation involves an electronic nose for heath care, which combines volatile organic component analysis and artificial neural networks for the detection of various diseases such as diabetes, cancer and other respiratory problems [145].

Even though the correlation of acetone content in exhaled breath with blood glucose concentration is highly dependent on external factors such as experimental conditions (glucose injection, insulin injection), the age, diet, health and physical activities of the patient, sample collection time, etc. [146], over the last decade many studies have reported positive [147,148,149,150], negative [148,151] and zero correlations [149,152,153] between acetone content and glucose concentration. The difficulty in obtaining a linear correlation is a major setback in this area, on which focused experiments are being carried out.

### 2.6. Blood-Based Glucose Sensors

Blood is a specialized bodily fluid that comprises plasma and cells that circulate across the entire body, supplying essential substances such as sugars, hormones, and oxygen around the body, acting as a powerhouse to detect and monitor health status [154]. Glucose detection was first initiated with blood-based sensors, and so far, blood is still the most used bodily fluid for diabetes detection. Blood glucose concentration has become the standard by which glucose content in other bodily fluids is correlated. Blood sugar levels for non-diabetic and diabetic patients range from 70 mg/dL to 140 mg/dL and 80 mg/dL to 180 mg/dL, respectively. As discussed earlier, the major drawbacks of this method are its invasiveness during sample collection and the associated pain, which is a serious issue for diabetic patients, as their wound healing rates are slow. However, it is still considered to provide the most accurate and trustworthy results [155,156].

**Figure 9:**
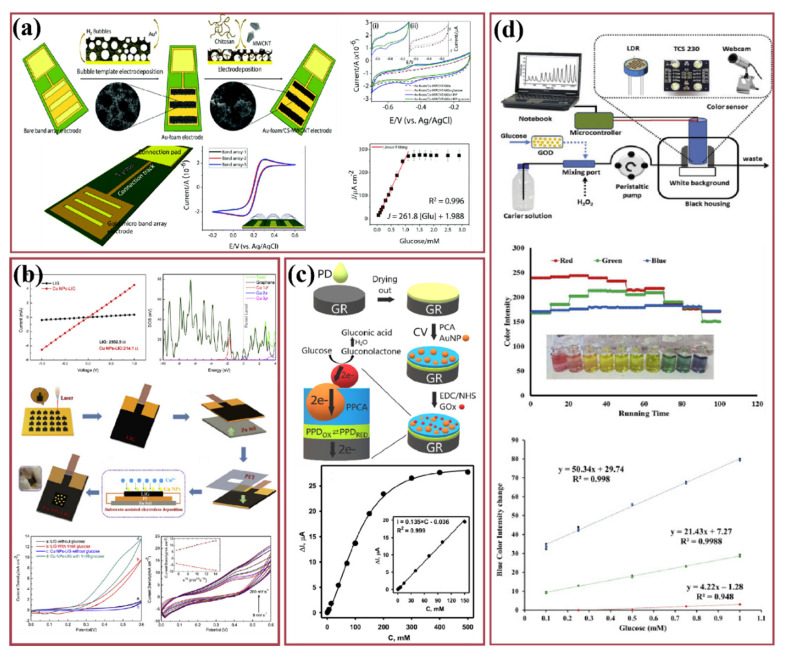
(**a**) Illustration of the 2-step electrochemical deposition process used to fabricate the Au-foam/CS–MWCNT electrode. (Reproduced with permission from Pemble et al., R. Soc. Chem., 2019 [157]. Copyright 2019, Royal Society Of Chemistry; O’reilly.) (**b**) Schematic illustration of fabrication process of the flexible Cu NPs-LIG sensor. (Reproduced with permission from Y. Zhang et al., Carbon, 2019 [164]. Copyright 2019, Elsevier Ltd.) (**c**) Working principle and operation of the GR/PPD/(AuNP)PPCA-GOx electrode biosensor. (Reproduced with permission from Minkstimiene et al., Microchem. J., vol.154, p. 104665, May 2020 [167]. Copyright 2020, Elsevier Ltd.) (**d**) The design of the color sensor in the flow analysis system. Color recognition device tested using a series solution (inset) at different pH values of 2.0 to 11.0 with the addition of universal pH solution. (Reproduced with permission from Fatoni et al., Sens. Bio-Sens. Res., pp. 30153–30159, 2020 [35]. Copyright 2020, authors under open access licenses).

The journey of portable blood glucose monitoring devices started from enzymatic biosensors. Juska et al. developed a dual-enzyme, micro-band array biosensor to analyze glucose concentrations in sterile human serum samples. This sensor was based on the electrodeposition of CNT/chitosan (CS) composite embedded in nanostructured Au-foams placed on microfabricated Au band electrodes. Here, foam was used to provide a larger surface area for electrodeposition of CS:MWCNT (Figure 9a). An enzyme mixture composed of GOx and HRP was immobilized on the nanocomposite surface. The obtained surface provided higher sensitivity (261.8 μA mM^−1^ cm^−2^) along with biocompatibility, conductivity, and chemical activity. The shelf life of these sensors was reported to be 45 days. Chronoamperometry was used to detect glucose in the linear range of 0.05 mM to 1.1 mM with an LOD of 0.025 mM [157].

Another study involved the development of an enzymatic glucose sensor by immobilizing GOx through the entrapment method in an anionic polymer with polypyrrole–poly(sodium- 4-styrenesulphonate) film. In general, as the sensing ability of enzymatic sensors is prone to change due to various factors such as temperature, pH, analyte concentration, etc., this study was sought to analyze the reproducibility and stability of the sensors. It was observed that the optimum temperature and pH were 25 °C and 8, respectively, and the LOD of this sensor was reported as 0.01–1000 μM [158]. In these devices, biofouling of the transducers and enzyme stability are issues; in order to address them, Marquez et al. developed a biosensor with a protective coating. The sensor was developed using electrodeposited calcium alginate hydrogel membrane with TMB as the mediator. Here, GOx and HRP were electrodeposited within a hydrogel network to improve enzymatic activity. The device showed a sensitivity of 0.27 µA cm^−2^ mM^−1^ in the linear working range of 2–12 mM with an LOD of 126 μM [159].

A study conducted by Canovas and team involved a wireless potentiometric paper-based sensing platform for glucose monitoring. Here, Nafion membrane-coated filter paper was used as the working electrode to entrap GOx, and a conductive paper with polyvinylbutyral-based membrane as the reference electrode. Detection of H_2_O_2_ generated due to the enzymatic reaction facilitated glucose monitoring. Selectivity achieved by the sensor in the range of 0.3–3 mM was −95.9 ± 4.8 mV/dec glucose [160]. A later study sought to tune the properties of the electrode by substituting the Nafion coating with a polyelectrolyte of the same family—Aquivion. The coatings served as semipermeable membranes towards interferents along with trapping GOx, which aided in increasing the sensitivity (−133.5 ± 9.9 mV/dec glucose) of the sensor towards H_2_O_2_. On substituting Nafion with Aquivion, the linear range jumped from 0.3–3 mM to 1–30 mM. This paper-based electrode biosensor was able to trap the enzyme and deflect the negatively charged interfering species found along with the analyte in entire blood samples [161].

In one studiy, Qasemi et al. exploited fluorescence properties by fabricating a fluorescence-active superabsorbent hydrogel. They used gum tragacanth nanoparticles, acrylic acid (AA) as monomer, and N,N’-methylenebisacrylamide (MBA) and fluorescein O,O′-diacrylate (FlA-DA) as cross-linkers. Cadmium telluride quantum dots (CdTe QDs) and GOx enzyme were entrapped within the cross-linked matrix of the obtained hydrogel using the swelling–diffusion method. Here, in the presence of CdTe QDs and FIA-DA, the electrode was quenched by H_2_O_2_ during the enzymatic reaction, which helped in achieving higher fluorescence intensity. The device showed an LOD of 0.1 mM and range of 0.1–10 mM with untreated whole blood samples [162].

With emphasis on production costs, a commercially viable technique of fused deposition modeling (a type of 3D printing) was used by Cardoso and group to develop a polylactic acid-containing graphene (G-PLA) based biosensing platform. The sensing capabilities of these platforms were due to the immobilization of oxidizing enzymes by the cross-linking agent glutaraldehyde, which was supported by the oxygenated groups present in the G-PLA polymeric matrix. The sensitivity of the platform was increased by mechanical polishing and immersion in solvents for surface treatment. This increase in the electrochemical activity of the sensor made it suitable for use in the detection of uric acid and nitrite content in blood alongside glucose. The LOD of this sensor was reported to be 15 μM with recovery values between 90–105% for the analysis of plasma. Considering the ease of fabrication, reduced amounts of waste in the fabrication process, and fast prototyping, these 3D-printed biosensing platforms can easily be produced on a large scale, making this fabrication technique highly desirable [163].

Owing to market demand for wearable sensors, the development of a nonenzymatic flexible electrochemical glucose biosensor was an attractive work by Zhang et al., who successfully developed a flexible enzyme-free glucose amperometric biosensor with a CuNP-anchored LIG composite through substrate-assisted electroless deposition (Figure 9b). The prepared Cu NPs-LIG sensor was reported to have a high sensitivity of 495 mA mM^−1^ cm^−2^ towards glucose with an LOD of 0.39 µM and faster response time of 0.5 s [164]. Azharudeen et al. used a thermal decomposition method to synthesis nanostructured NiO along with PANI. In this non-enzymatic biosensor, NiO nanocomposites were modified with different compositions of PANI and comparisons of the catalytic activities of these nanocomposites were drawn. It was reported that the NiO/6% PANI showed superior catalytic ability, which was explained by the increased surface area leading to better electron transfer. The obtained sensors had sensitivity of 606.13mA mM^-1^ cm^-2^ with an LOD of 0.19 μM [165]. Balasubramanian et al. fabricated thin nanosheets of cobalt hydroxide rich in Co vacancies. The vacancy defects helped improve the active sites and electron transfer rates, which intensified the electrocatalytic oxidation of glucose. The range of detection was reported as 0.4 μM–8.23 mM, while the LOD was 295 nM [166].

Minkstimiene et al. produced a biosensor with sensitivity and anti-interference towards common analytes such as uric acid and acetylsalicylic acid. This nanocomposite-based sensor was prepared by absorbing 1,10-phenanthro- line-5,6-dione (PD) on a graphite rod electrode, which was then coated with pyrrole-2- carboxylic acid (PCA) and colloidal AuNP through electrochemical polymerization of PD and PCA, with encapsulation of AuNPs in the poly(pyrrole-2-carboxylic acid) (PPCA) matrix. The GOx was immobilized by the carboxylic groups present in PPCA through covalent bonding. The working principle and operation are illustrated in Figure 9c. The prepared sensor showed a linear range of 0.2–150.0 mM and sensitivity 0.135 μA/mM with an LOD of 0.08 mM [167].

A colorimetric real-time glucose detection sensor developed using chitosan cryogel beads was another innovation in this field. In this biosensor, glucose quantification in blood was by color recognition performed using three different components: a light-dependent resistor (LDR), a TCS230 commercial photodetector, and a webcam. Here, the chitosan cryogel bead biosensing element was used to immobilize GOx in a crosslinked network of glutaraldehyde. Detection was based on the formation of yellow color upon H_2_O_2_ and titanium oxysulfate reaction. The RGB color intensity was studied and correlated against the glucose concentration using a software called color sensor reader, illustrated in Figure 9d. A detection range of 0.1 mM to 2.5 mM in diluted blood samples with an LOD of 0.14 mM was reported [35]. The performance of various blood-based glucose sensors is summarized in Table 5.

## 3. Conclusions

Abundant research and numerous publications on biofluid-based glucose sensors reflect the potential and importance of this research field in the present-day scenario. Glucose monitoring technologies have prevailed in the management of diabetes for decades now. Continuous glucose sensors are becoming a critical component of the insulin delivery system together with being selective, sensitive, rapid, reliable, and acceptable for continuous patient use. Various methods (such as electrochemical, optical, spectroscopic and microneedle transdermal techniques) with aspects of their functional and technological advancements, along with material exploitation, have been discussed in this review. Some of the crucial advances include (1) achieving linearity in device response to varying glucose concentrations in different types of biofluids and corelating them with blood glucose level; (2) utilizing novel materials and device structures to the maximum extent for their sensing abilities; (3) discovering new modalities with less complications; (4) integration of electronic technologies into the biosensing platforms; (5) improved device lifetimes (up to 85 days); (6) economical product designs; and, (7) multi-fluid-based sensing capabilities. These advances notwithstanding, and despite the huge developments of the past decade, there still exist a few practical problems associated with biofluid-based sensors that have influenced and slowed down the overall evolution of such devices. Some of the major existing problems include (1) scalability of the device for mass production; (2) portability for practical usage; (3) limited shelf life; (4) loss of device sensitivity owing to the protective layer during real-time monitoring; (5) corrosion, toxicity and biocompatibility of implantable devices; (6) higher power consumption for faster response; (7) analyte contamination; (8) necessity of personalization; and (9) reliability in hands-on application compared to laboratory-scale devices. Apart from these, specific biofluid-based sensors also have individual issues. One such issue involves low glucose concentrations in analytes such as saliva, tears and sweat, posing difficulties during correlation with blood glucose levels, a task that requires high sensitivity and lower detection limits for these devices. Urine is a complex analyte, which makes it difficult to differentiate between hyperglycemia and other health conditions such as renal tubular disorder, pregnancy, patients with low renal thresholds for glucose, renal dysfunction, etc. Detection and quantification are other hurdles for hypoglycemic patients due to the absence of glucose content in analytes such as urine, breath, and tears. Insufficient amounts of sample generation are a common complication faced with sweat, tears and urine, which require further mechanical and chemical simulation such as the introduction of foreign bodies or iontophoresis for ease of sample collection. However, unstimulated sample collection is always preferred over any simulated method due to discomforts such as irritation, damage to the conjunctiva, or alterations to the original glucose concentration in analytes, which in turn can cause changes in the correlations with the glucose concentration in blood. Contact lenses with enzymatic coatings and chips are remarkable pieces of engineering that have helped in eliminating painful tear collection methods, but at the same time, they are still not commercially viable due to the issue of dry eyes. On the other hand, although breath is a promising gaseous non-invasive analyte for glucose monitoring, breath-based sensors present major drawbacks for data analysis and the selection of suitable biomarkers.

In the near future, the research focus will be on tackling the shortcomings mentioned above and rigorous research aimed at obtaining crucial information to aid timely treatment is being emphasized with the help of CGM. Various advanced techniques, such as screen printing, MEMS, and the fabrication of paper-based, microscale, in vivo, micropatterned, computational nano/micro devices, etc., are being utilized to develop high-tech glucose sensors. Barring limitations to the extent of evolution in this field, such glucose monitoring techniques can also be exploited for far-reaching applications in areas such as the monitoring and control of artificial organs.

## Figures and Tables

**Table 1:** The performance of various saliva-based glucose sensors.

Glucose Sensors	Method of Detection	Biological Fluid Used	Linear Detection Range	Limit of Detection	References
CuO/PCL@PPy/ITO	Electrochemistry	Saliva	2 µM–6 mM	0.8 µM	[44]
Co_3_O_4_ needles on Au honeycomb	Electrochemistry	Saliva	20–100 µM	20 µM	[60]
rGO modified Nb_2_O_5_	Electrochemistry	Tears, Urine, Saliva	1–10 mM	1 mM	[62]
Nanoporous palladium(II) bridged coordination polymer	Colorimetry	Tears Saliva	0–47 nM	61 nM 91 nM	[63]
Pt/Ni@NGT paper based device	Colorimetry	Tears, Saliva	0.1–50 mM	1 pM	[64]
PG/Co(OH)_2_	Chemiluminescence	Tears, Saliva	3.0 × 10^−9^–4.0 × 10^−5^ mol L^−1^	6.4 × 10^−10^ mol L^−1^	[65]
Flexible OECTs- GOx-GO/PANI/Nafion-graphene/Pt	Electrochemistry	Saliva	0.1 µM–1 mM	0.01 µM	[48]
IrO_2_@ NiO nanowires	Electrochemistry	Saliva	0.5 μM–2.5 mM	0.31 µM	[57]
PEDOT:PSS with Ni/Al LDH	Electrochemistry	Saliva	0.1–8.0 mM	0.02 mM	[51]
AgNPs/MoS_2_	Electrochemistry	Saliva, Sweat	0.1–1000 µM	0.03 µM	[58]
Hb deposited LPG	Electrochemistry	Saliva	0.05–2 mmol/L	0.1 mmol/L	[56]

**Table 2:** The performance of various tear-based glucose sensors.

Glucose Sensors	Method of Detection	Biological Fluid Used	Linear Detection Range	Limit of Detection	References
rGO modified Nb_2_O_5_	Electrochemistry	Tears, Urine, Saliva	1–10 mM	1 mM	[62]
Nanoporous palladium(II) bridged coordination polymer	Colorimetry	Tear Saliva	0–47 nM	61 nM 91 nM	[63]
Pt/Ni@NGT paper- based device	Colorimetry	Tears, Saliva	0.1–50 mM	1 pM	[64]
PG/Co(OH)_2_	Chemiluminescence	Tears, Saliva	3.0 × 10^−9^–4.0 × 10^−5^ mol L^−1^	6.4 × 10^−10^ mol L^−1^	[65]
CdSe/ZnS donor, malachite green dextran acceptor on ZnO nanorods-silicon hydrogel lens	Fluorescence resonance energy transfer	Tears	0.03–3 mmol/L	0.03 mmol/L	[72]
GOx-CHIT/Co_3_O_4_ /Au	Electrochemistry	Tears	0–100 nM	100 nM	[73]
Pt/Ir wire with selective layer of Nafion and 1,3-diaminobenzene	Electrochemical/colorimetry	Tears	0–40 mM	1 µM	[75]
PS-GCCA on RGP contact lens	Colorimetry/diffraction spectrometry	Tears	0–50 mM	0.05 mM	[76]
PS-MCC	Colorimetry/Diffraction spectroscopy	Tears, Blood	0–20 mM	20 mM	[74]
COOH chitosan- functionalized NG	Electrochemistry	Tears	0–12 mM	9.5 µM	[78]

**Table 3:** The performance of various urine-based glucose sensors.

Glucose Sensors	Method of Detection	Biological Fluid Used	Linear Detection Range	Limit of Detection	References
rGO modified Nb_2_O_5_	Electrochemistry	Tears, Urine, Saliva	1–10 mM	1 mM	[62]
PMBA@Au/optical fiber with AET/AuNPs	SPR	Urine	8 × 10^−8^–5 × 10^−2^ M	0.8 µM	[81]
GO decorated AuNBs@Ag	Raman spectroscopy/SPR	Urine	0.01–10^−4^ µM	80 nM	[82]
2D Triangular photonic crystal structure	Photonic band gap	Urine	0 gm/dL to 10 gm/dL		[84]
N,B doped CNPs	Fluorescence	Urine	0–900 µM	1.8 µM	[91]
Fe_3_O_4_–Fe(OH)3@GOx–polyDA	Colorimetry/UV Spectroscopy	Urine	5–500 µM	3 µM	[94]
Ag^+^NPs @ cotton fabric	Electrochemistry	Urine	100–2000 µM	80 µM	[95]
Fe-Pd/rGO	Colorimetry/UV spectrometry	Urine	0–200 µM	1.76 µM	[96]
Cu-MOF	Electrochemistry	Urine	0.06 μM–5 mM	10.5 nM	[98]
Bi_2_Fe_3_-few layers modified GC electrodes	Electrochemistry	Urine	10–100 µM	6.1 µM	[99]
CoFe@N-Graphene	Electrochemistry	Urine	0–3.25 mM	37.7 µM	[100]

**Table 4:** The performance of various sweat- and interstitial fluid-based glucose sensors.

Glucose Sensors	Method of Detection	Biological Fluid Used	Linear Detection Range	Limit of Detection	References
5-layered microfluidic chip	Colorimetry	Sweat	0.1–0.5 mM	0.03 mM	[36]
AgNPs/MoS_2_	Electrochemistry	Saliva, Sweat	0.1–1000 µM	0.03 µM	[58]
PET-based Au electrodes	Electrochemistry	Sweat	0.02–1.11 mM	2.7 µM	[104]
Carbon graphite ink on adhesive tapes	Electrochemistry	Sweat	0.48–2.59 mM	0.80 µM	[105]
PEDOT:PSS/LIG	Electrochemistry	Sweat	10 μM–9.2 mM	3 µM	[107]
SBP_thi_ nanosheets	Electrochemistry	Sweat	0.82 μM–4.0 mM	0.27 µM	[108]
3D-PMED	Electrochemistry	Sweat	0–1.9 mM	5 µM	[114]
PVDF/Nafion/PtNPs/PANI nanofiber needle electrodes	Electrochemistry	Interstitial fluid	0–20 mM		[121]
Au/Au-multiwalled carbon nanotubes (MWCNTs)/ poly-methylene blue (pMB)/FADGDH electrodes	Electrochemistry	Interstitial fluid	0.05–5 mM	7 μM	[122]
COC-PPY polymer needle/Au/pTCA-GOx/Nafion	Electrochemistry	Interstitial fluid	0.05–20.0 mM	19.4 (±0.62) μA	[123]
Hard-PDMS / PI / Au/rGO/GOx/ Nafion electrodes	Electrochemistry	Interstitial fluid	0–30 mM	0.198 µM	[124]
Au/GO/AuNPs	Electrochemistry	Sweat	0.05–42 mM	12 µM	[133]

**Table 5:** The performance of various blood-based glucose sensors.

Glucose Sensors	Method of Detection	Biological Fluid Used	Linear Detection Range	Limit of Detection	References
Chitosan cryogel beads with TCS230	Colorimetry	Diluted blood	0.1–2.5 mM	0.14 mM	[35]
PS-MCC	Colorimetry/Diffraction spectroscopy	Tears, Blood	0–20 mM		[80]
Au foam@ CNT-modified Chitosan	Electrochemistry	Blood serum	0.05–1.1 mM	0.025 mM	[157]
Polypyrrole–poly (sodium- 4 -styrenesulphonate) film	Electrochemistry	Blood	1 × 10^−8^–1 × 10^−3^ M		[158]
Calcium alginate hydrogel membrane	Electrochemistry	Whole blood	2–12 mM	126 µM	[159]
Nafion/Aquivion-coated Pt electrodes	Potentiometric Electrochemistry	Whole blood	0.3–3 mM		[160]
GT/AA/MBA/FlA-DA/ CdTe quantum dots-Hydrogel	Fluorescence	Whole blood	0.1–10 µM	0.1 µM	[162]
G-PLA	Electrochemistry	Blood	0.5–250 μmol L^−1^	15 μmol L^−1^	[163]
NiO/PANI	Electrochemistry	Blood serum	0–100 µM	0.19 µM	[165]
V_Co_-Co(OH)_2_ nanosheets	Electrochemistry	Blood serum	0.4 μM–8.23 mM	295 nM	[166]
PD/PPCA/AuNPs/Graphite electrode	Electrochemistry	Blood serum	0.2–150 mM	80 µM	[167]

## Data Availability

Not applicable.
